# On the potential of models for location and scale for genome-wide DNA methylation data

**DOI:** 10.1186/1471-2105-15-232

**Published:** 2014-07-03

**Authors:** Simone Wahl, Nora Fenske, Sonja Zeilinger, Karsten Suhre, Christian Gieger, Melanie Waldenberger, Harald Grallert, Matthias Schmid

**Affiliations:** 1 Research Unit of Molecular Epidemiology, Helmholtz Zentrum München – German Research Center for Environmental Health, Ingolstädter Landstrasse 1, 85764 Neuherberg, Germany; 2 Institute of Epidemiology II, Helmholtz Zentrum München – German Research Center for Environmental Health, Ingolstädter Landstrasse 1, 85764 Neuherberg, Germany; 3 German Center for Diabetes Research (DZD), Ingolstädter Landstrasse 1, 85764 Neuherberg, Germany; 4 Department of Statistics, Ludwig-Maximilians-Universität München, Ludwigstrasse 33, 80539 München, Germany; 5 Department of Physiology and Biophysics, Weill Cornell Medical College in Qatar (WCMC-Q), Qatar Foundation - Education City, P.O. Box 24144 Doha, Qatar; 6 Institute of Bioinformatics and Systems Biology, Helmholtz Zentrum München –German Research Center for Environmental Health, Ingolstädter Landstrasse 1, 85764 Neuherberg, Germany; 7 Institute of Genetic Epidemiology, Helmholtz Zentrum München – German Research Center for Environmental Health; 8 Institute of Medical Biometry, Informatics and Epidemiology, Rheinische Friedrich-Wilhelms-Universität, Sigmund-Freud-Str. 25, 53127 Bonn, Germany

**Keywords:** DNA methylation, Beta regression, GAMLSS, Infinium HumanMethylation450k BeadChip, EWAS, Modeling variability, Resampling, Model performance, Model comparison, Models for location and scale

## Abstract

**Background:**

With the help of epigenome-wide association studies (EWAS), increasing knowledge on the role of epigenetic mechanisms such as DNA methylation in disease processes is obtained. In addition, EWAS aid the understanding of behavioral and environmental effects on DNA methylation. In terms of statistical analysis, specific challenges arise from the characteristics of methylation data. First, methylation *β*-values represent proportions with skewed and heteroscedastic distributions. Thus, traditional modeling strategies assuming a normally distributed response might not be appropriate. Second, recent evidence suggests that not only mean differences but also variability in site-specific DNA methylation associates with diseases, including cancer. The purpose of this study was to compare different modeling strategies for methylation data in terms of model performance and performance of downstream hypothesis tests. Specifically, we used the generalized additive models for location, scale and shape (GAMLSS) framework to compare beta regression with Gaussian regression on raw, binary logit and arcsine square root transformed methylation data, with and without modeling a covariate effect on the scale parameter.

**Results:**

Using simulated and real data from a large population-based study and an independent sample of cancer patients and healthy controls, we show that beta regression does not outperform competing strategies in terms of model performance. In addition, Gaussian models for location and scale showed an improved performance as compared to models for location only. The best performance was observed for the Gaussian model on binary logit transformed *β*-values, referred to as *M*-values. Our results further suggest that models for location and scale are specifically sensitive towards violations of the distribution assumption and towards outliers in the methylation data. Therefore, a resampling procedure is proposed as a mode of inference and shown to diminish type I error rate in practically relevant settings. We apply the proposed method in an EWAS of BMI and age and reveal strong associations of age with methylation variability that are validated in an independent sample.

**Conclusions:**

Models for location and scale are promising tools for EWAS that may help to understand the influence of environmental factors and disease-related phenotypes on methylation variability and its role during disease development.

## Background

DNA methylation is an important epigenetic mechanism that is involved in the regulation of gene expression [1]. In humans, methylation occurs most frequently at cytosine (C) nucleotides preceding a guanine (G) nucleotide, referred to as CpG sites [2]. Owing to its role in numerous physiological processes and disease states including cancer, changes in DNA methylation introduced by lifestyle factors and disease-related phenotypes have received increasing attention [3-9]. High-throughput array technologies such as the Illumina Infinium HumanMethylation450k BeadChip [10] have enabled epigenome-wide association studies (EWAS) to explore the relationship between phenotypes and DNA methylation in large population-based studies [2].

The statistical analysis of genome-wide DNA methylation data entails specific challenges. Most commonly, methylation signals from the Infinium HumanMethylation450K BeadChip are summarized as *β*-values, the proportion of methylated in relation to the sum of methylated and unmethylated signals at a specific genomic site in a biological sample [10]. Methylation *β*-values are bounded in the unit interval, with values occuring between 0 (corresponding to 0% methylation at the respective site) and 1 (corresponding to 100*%* methylation at the respective site). As typical for proportion data, their distributions display substantial heteroscedasticity [11]. Specifically, they tend to show smaller variances when located near the boundaries 0 and 1 as compared to the center of the unit interval. In addition, their distributions are typically skewed. Therefore, when using *β*-values as response variable in a statistical regression model, traditional modeling strategies that assume a normally distributed response might be inappropriate [12]. To solve this problem, it has been proposed to use the less heteroscedastic *M*-values for Gaussian regression, which are approximately equal to binary logit transformed *β*-values [13]. An alternative transformation for proportion data, which is often used in ecology to achieve variance stabilization, is the arcsine square root transformation [14]. This transformation has also recently been applied to methylation data [15]. In addition, *beta regression*, a statistical regression technique that is tailored to bounded response variables, has been proposed as a “natural” modeling strategy for proportion data [11,16]. Currently beta regression is beginning to find application in methylation data analysis [17-20]. Although improved performance of beta regression as compared to traditional approaches was reported [18], a thorough comparison of these competing approaches for the purpose of univariate screening for genome-wide phenotype-methylation associations is lacking.

Furthermore, there is increasing evidence that not only mean differences but also variability of methylation plays a role in disease processes, including cancer [21-25]. This enhances the need to screen for an effect of lifestyle factors and disease-related phenotypes on DNA methylation level and variability [9,26,27]. In these studies, variability was either modeled isolated from the mean, in a combined test together with the mean, or not explicitly modeled as a function of covariates at all.

Here we propose *generalized additive models for location, scale and shape* (GAMLSS) [28] as a flexible approach to model methylation data. GAMLSS can be specified such that a regression model is estimated for the mean (location parameter), and another model for the variability (scale parameter), so that covariate effects on both parameters can be quantified simultaneously. Joint modeling of location and scale is preferable to separate modeling since mean and variance of bounded *β*-values are not independent [11].

The purpose of this study was to compare model performance as well as the performance of downstream hypothesis tests of (1) beta regression as compared to Gaussian regression on raw, binary logit or arcsine square root transformed *β*-values, (2) with and without simultaneous modeling of covariate effects on the scale parameter within the GAMLSS framework. Using simulated and real data sets from the large population-based research platform Cooperative Health Research in the Region of Augsburg (KORA) (*n*=2299), we demonstrate that models for location and scale, specifically the Gaussian model on *M*-values, increase predictive performance as compared to models for location only, while being more sensitive towards violations of the distribution assumption and towards the presence of outliers in the methylation data. To address this problem, we propose and evaluate a resampling-based strategy based on parametric bootstrapping followed by rank-based reassignment of the original data. All findings concerning model performance and evaluation of the resampling-based strategy are subsequently validated in an independent data set of acute lymphoblastic leukemia patients and healthy controls. Finally, an application in an EWAS of BMI and age is presented.

## Methods

### Data sets

Our study is based on two large data sets of 1799 and 500 subjects from the F4 and F3 studies, respectively, of the population-based research platform KORA [29]. These data sets have been used in published EWAS before [3-5], and study design and data collection have been described in detail [29,30]. Methylation measurements from whole blood were obtained from the Illumina Infinium HumanMethylation450K BeadChip [10], as described in [3,4]. Raw methylation data were preprocessed and quality controlled as described in Additional file 1. Subjects with missing or outlying (value outside mean ± 5 standard deviations) covariate information were excluded from the analysis, so that the final F4 methylation data set comprised 413,641 autosomal sites, mainly CpG sites, and 1763 subjects. Similarly, data from 486 F3 subjects were available for analysis. A phenotypic description of both data sets is provided in Tables S1 and S2 in Additional file 2. For the F4 data, genome-wide single nucleotide polymorphism (SNP) data were obtained using the Affymetrix GeneChip array 6.0, and genotypes were imputed with IMPUTE v0.4.2 using the HapMap2 reference panel [31]. In the F3 population, genotyping was performed with the Illumina HumanOmniExpress BeadChip and the Illumina 2.5 BeadChip, followed by imputation with IMPUTE v2.3.0 using the 1000g phase1 reference panel (integrated haplotypes).

### Modeling strategies for DNA methylation data

The Illumina Infinium HumanMethylation450K BeadChip assay provides a readout of methylated and unmethylated signal intensities from hybridization of DNA fragments from a biological sample to oligonucleotides attached to beads on the chip. For a given CpG site *j*, the methylated (*M*_*j*_) and unmethylated (*U*_*j*_) signal intensities are combined to methylation *β*-values [13]:

$$\beta {\text{-value}}_{j}=\frac{\text{max}(M_{j},0)}{\text{max}(M_{j},0)+\text{max}(U_{j},0)+\alpha_{\beta }}$$

where the inclusion of an offset *α*_*β*_=100 is recommended as as a stabilization in the situation when both methylated and unmethylated signal intensities are small [13]. Since the total number of signals exceeds 1000 in the majority of CpG sites, the offset does not induce much bias. By definition, *β*-values are bounded to the unit interval. Ignoring the offset, they can be interpreted as proportion of methylation at a specific CpG site in a biological sample. Typically, *β*-values at specific CpG sites tend to have a skewed distribution, centered at either a low or a high methylation state, with variances smallest for CpG sites centered close to the bounds of the standard unit interval (Figure S1 in Additional file 3).

We consider regression models with *β*-values as response variable, and disease-related phenotypes as explanatory variables. When modeling proportions as a function of covariates, their boundedness as a function of covariates, their boundedness implies that the conditional mean must be a nonlinear function of the explanatory variables, since a linear combination of the explanatory variables would not be restricted to values within (0,1). In addition, it implies that the conditional variance must be a function of the conditional mean, i.e. the distribution is heteroscedastic [32]. Both conditions are not met when Gaussian regression is used, so that both the expectation and the variance structure are misspecified, potentially resulting in biased and inconsistent estimation.

To overcome these problems, it was proposed to use the *log*_2_ ratio of the methylated to unmethylated signal intensities:

$$M{\text{-value}}_{j}={\text{log}}_{2}\left(\frac{\text{max}\;\left(M_{j},0\right)+\alpha_{M}}{\text{max}\;\left(U_{j},0\right)+\alpha_{M}}\right)\;,$$

where *α*_*M*_=1 is typically used. *M*-values are defined on (-*∞*,+*∞*) and are less heteroscedastic than *β*-values [13]. They are also more symmetric than *β*-values (Figure S1 in Additional file 3). Ignoring the offsets *α*_*β*_ and *α*_*M*_, the *M*-value is approximately equal to the binary logit of the *β*-value:

$$M{\text{-value}}_{j}\approx {\text{log}}_{2}\;\left(\frac{\beta {\text{-value}}_{j}}{1-\beta {\text{-value}}_{j}}\right)\;.$$

When using binary logit transformed *β*-values as response variable in Gaussian regression, the corresponding model is specified as follows: Let ***y***=(*y*_1_,…,*y*_*n*_)^*T*^ be the vector of *β*-values corresponding to *n* independent observations, and *h*(·) the binary logit transformation. Then the transformed response $\tilde{y}={\left(h(y_{1}),h(y_{2}),\ldots ,h(y_{n})\right)}^{T}$ is modeled as a function of covariates:

$$\begin{array}{l} \;\tilde{y}=X\gamma +\varepsilon ;\varepsilon ∼N\left(0,\sigma^{2}I\right)\;\\ E(\tilde{y})=X\gamma \; \end{array}$$

where **X** represents the *n*×*p* matrix of covariate values, ***γ***=(*γ*_1_,*γ*_2_,…,*γ*_*p*_)^*T*^ a vector of regression coefficients corresponding to *p* covariates, ***ε*** the *n*×1 vector of independently normally distributed error terms with variance *σ*^2^, and *E*(·) the expectation. Note that this model formulation also comprises Gaussian regression with untransformed *β*-values as response variable. In that case h(.) would be the identity function. In addition, the model can be extended to facilitate explicit modeling of *σ* as a function of covariates (see below).

In a titration experiment by Du *et al.*[13], *M*-values outperformed *β*-values in terms of power and precision when identifying differentially methylated sites based on methylation differences between two groups. However, when differential methylation was defined based on test statistics, superiority of *M*-values was only observed in small sample sizes, when regularized *t*-statistics were used [33]. In multivariate feature selection, *β*-values tended to better preserve the correlation structure of methylation signals [33]. Generally, due to the logit transformation, the interpretation of a regression model based on *M*-values is less intuitive than the interpretation of a corresponding model based on untransformed *β*-values, since covariate effects have to be interpreted with regard to the expectation of the transformed response $\tilde{y}$ rather than ***y*** itself.

In ecological applications, proportions are often transformed using the arcsine square root transformation

$$A{\text{-value}}_{j}={\text{sin}}^{-1}\left(\sqrt{\beta {\text{-value}}_{j}}\right)$$

to achieve homoscedasticity [14]. The transformed values are then for instance modeled with a Gaussian regression model as described above, specifying *h*(·) as the arcsine square root transformation. However, the arcsine square root transformation maps (0,1) to (0,*π*/2), so that values are still bounded.

A natural alternative to the Gaussian models considered above is beta regression, which explicitly takes into account that *β*-values are proportions [11,12,34,35]. The beta distribution has the density function

$$\begin{array}{ll} f\left(y;\mu ,\sigma \right)= & \frac{\Gamma \;\left(\frac{1}{\sigma^{2}}-1\right)}{\Gamma \;\left(\left(\frac{1}{\sigma^{2}}-1\right)\mu \right)\Gamma \;\left(\left(\frac{1}{\sigma^{2}}-1\right)\;(1-\mu )\right)}\cdot \;\\ & y^{\left(\frac{1}{\sigma^{2}}-1\right)\mu -1}\cdot {\left(1-y\right)}^{\left(\frac{1}{\sigma^{2}}-1\right)\;(1-\mu )-1},\; \end{array}$$

where *y*∈(0,1) represents the response vector, *μ*∈(0,1) a location parameter, *σ*∈(0,1) a scale parameter, and *Γ*(·) the gamma function. Typically, *f*(*y*;*μ*,*σ*) is skewed (see e.g. [11] for an illustration). Expectation and variance of a beta distributed random variable *y* are given by *E*(*y*)=*μ*, and*Var*(*y*)=*μ*(1-*μ*)*σ*^2^. Thus, beta regression incorporates boundedness and skewness of a proportion response [11]. Also, because *Var*(*y*) explicitly depends on *μ*, heteroscedasticity of the response can be modeled via beta regression.

The beta regression model is defined analogously to a generalized linear model and is formally given by

g(μ)=g(E(y))=Xγ

where ***μ*** represents the mean of a beta distributed response vector ***y***, and *g*(·) a strictly monotonic and twice differentiable link function that maps the open unit interval into [16]. In practice, the logit function is commonly chosen for *g*(·). Note that in case of *y*∈[0,1], values can be rescaled to lie in (0,1) [11]. In our methylation data, zeros did not occur after preprocessing.

To model the effect of the explanatory variables on *Var*(*y*) (and thereby the heteroscedasticity of the response explicitly), a “variable scale beta regression model” has been proposed as an extension. Thereby, location (***μ***) and scale (***σ***) parameter are both modeled, with a possibly distinct set of covariates [11].

Despite these appealing properties and modeling options, beta regression has been considered in few studies on DNA methylation data so far [17-20]. A thorough examination of its performance in comparison to competing modeling strategies, and specifically the relevance of modeling the scale parameter in beta and Gaussian regression models, has not been conducted.

### Generalized additive models for location, scale and shape (GAMLSS)

GAMLSS provides a flexible modeling framework for responses from a large class of distributions, including Gaussian and beta distributions [28,36]. It extends generalized linear and additive models first, by relaxing the distribution assumption, and second, by allowing for modeling not only the mean (location parameter) but also other distribution parameters (e.g. a scale parameter) as a function of explanatory variables. Consequently, beta regression is a special case of a GAMLSS. Moreover, the GAMLSS methodology allows for modeling the residual error variance *σ*^2^ of the Gaussian models described above. Together, this makes GAMLSS highly relevant for modeling DNA methylation data. Specifically, it provides a unique framework for the comparison of the different approaches to model methylation data considered in this study.

In this article we focus on the fully parametric GAMLSS with linear covariate effects and two submodels:

$$\begin{array}{lll} & g_{1}(\mu )=X_{1}\gamma_{1}\; & \;\\ & g_{2}(\sigma )=X_{2}\gamma_{2}\; \end{array}$$

where *X*_1_ and *X*_2_ are matrices of covariate values, ***γ***_1_ and ***γ***_2_ the respective vectors of regression coefficients of lengths *p*_1_ and *p*_2_, and ***μ*** and ***σ*** the location and scale parameters as defined above within the context of Gaussian and beta regression. In “fixed scale models”, i.e. models for location only (as opposed to “variable scale models”), *X*_2_ reduces to an all-ones vector, and ***γ***_2_ to a scalar intercept. For beta regression models, the link functions *g*_1_(·) and *g*_2_(·) were both chosen as the natural logit. For Gaussian regression models based on raw *β*-values, *M*-values and *A*-values, they were chosen as identity and log links, respectively.

### Analysis of model fit and predictive performance

Model comparisons were conducted on the KORA F4 data set (n = 1763). We compared eight different modeling strategies: Four fixed scale models, i.e. Gaussian regression on raw data (ra), on binary logit transformed data (lo) and on arcsine square root transformed data (ar), as well as beta regression (be), and the corresponding four variable scale models (ra+, lo+, ar+ and be+) that additionally regressed the scale parameter on the covariates (Table 1). The variable scale models and the fixed scale beta regression model were fitted as GAMLSS (R package *gamlss*, version 4.2-6) [28,36], whereas the remaining fixed scale models were fitted as generalized additive models (GAMs) (R package *mgcv*, version 1.7-24) [37].

**Table 1:** Competing models for methylation data analysis

**Model (abbreviation)**	**Transformation**	**Distribution**	**Submodels**
	**of response y**		
*Fixed scale models*			
raw (ra)	y	Gaussian	*μ* only
logit2 (lo)	$\underset{2}{\log}\left(\frac{y}{1-y}\right)$	Gaussian	*μ* only
arcsine (ar)	$\overset{-1}{\sin}(\sqrt{y})$	Gaussian	*μ* only
beta (be)	y	beta	*μ* only
*Variable scale models*			
raw+ (ra+)	y	Gaussian	*μ* and *σ*
logit2+ (lo+)	$\underset{2}{\log}\left(\frac{y}{1-y}\right)$	Gaussian	*μ* and *σ*
arcsine+ (ar+)	$\overset{-1}{\sin}(\sqrt{y})$	Gaussian	*μ* and *σ*
beta+ (be+)	y	beta	*μ* and *σ*

In location and scale submodels, the respective parameter was specified as a linear function of covariates that are known to have an effect on methylation levels, including age [6], sex [38], smoking state (with the categories current, never and former smoker) [3], alcohol intake (in g/d) [8], physical activity (with the categories active and inactive) [7], body mass index (BMI) [5,9], white blood cell count (WBC) and estimated proportions of six white blood cell types as derived using the method by Houseman *et al.*[39]. In the location submodel, the first 15 control probe principal components (PCs) were included to avoid technical confounding (see Additional file 1).

Model performance was investigated in a random set of 10,000 CpG sites. Repeating analyses in newly drawn sets of CpG sites showed that results were stable towards the CpG sites chosen. We fitted every model to a random subsample comprising 50% of the observations as a training set, drawn anew for each CpG site, with the remaining 50% left out as a test set. The pseudo R ^2^ criterion [40,41] was then calculated from both training and test sets as a measure of model fit and predictive model performance, respectively. The pseudo R ^2^ criterion has been used to compare beta and Gaussian regression before [34,35]. It measures the improvement of the likelihood of the fitted as compared to the intercept-only model, and is therefore more appropriate in the context of models for location and scale than measures based on correlation or deviance of fitted versus observed response values, since it better accounts for the fit of the scale submodel. The precise definition of the pseudo R ^2^ criterion for the different models is given in Additional file 1. The analysis was repeated in a smaller sample size of 250 observations, randomly drawn for each CpG site, to evaluate the impact of sample size on model performance.

Residual model fit was assessed using Shapiro-Wilk tests on normalized quantile residuals [28].

### Simulation study

To assess type I error and power of downstream hypothesis tests of the competing models, and the sensibility towards violations of the distribution assumptions, a data-driven simulation study was conducted comprising beta distributed and real-data distributed methylation responses. All data were deliberately simulated to be close to the observed methylation data, in terms of average location and scale, as well as in terms of the size of observed covariate effects, aiming to approximate the real-data scenario as closely as possible.

#### Beta-distributed data setting

For each CpG site *j*, *j*=1,…,10000, real methylation data were modeled using variable scale beta regression with covariates as described above. From these models, the fitted distribution parameter vectors ${\hat{\mu }}_{j}=g_{1}^{-1}\left(X_{1}{\hat{\gamma }}_{j1}\right)$ and ${\hat{\sigma }}_{j}=g_{2}^{-1}\left(X_{2}{\hat{\gamma }}_{j2}\right)$ were obtained, where ${\hat{\gamma }}_{j1}$ and ${\hat{\gamma }}_{j2}$ represent vectors of the estimated covariate effects. Then, the entries of ${\hat{\gamma }}_{j1}$ and ${\hat{\gamma }}_{j2}$ corresponding to the effects of one covariate, BMI, were manipulated to values given in Table 2, to represent zero, moderate and strong covariate effects on location and scale. Thereby, the moderate effect size was chosen such that observed effects of age, sex, smoking state, alcohol intake and physical activity, BMI and WBC were rarely stronger in absolute terms.

**Table 2:** Simulation settings

**Setting**	**Tested parameter**	$\gamma_{\text{BMI}}^{\left(\mu \right)}$	$\gamma_{\text{BMI}}^{\left(\sigma \right)}$
*Settings for the assessment of type I error*			
*μ*|-*σ*	$\gamma_{\text{BMI}}^{\left(\mu \right)}$	**0**	0
*μ*|+*σ* moderate		**0**	0.005
*μ*|+*σ* strong		**0**	0.05
*σ*|-*μ*	$\gamma_{\text{BMI}}^{\left(\sigma \right)}$	0	**0**
*σ*|+*μ* moderate		0.005	**0**
*σ*|+*μ* strong		0.05	**0**
*Settings for the assessment of power*			
*μ*|-*σ*	$\gamma_{\text{BMI}}^{\left(\mu \right)}$	**0.005**	0
*μ*|+*σ* moderate		**0.005**	0.005
*μ*|+*σ* strong		**0.005**	0.05
*σ*|-*μ*	$\gamma_{\text{BMI}}^{\left(\sigma \right)}$	0	**0.005**
*σ*|+*μ* moderate		0.005	**0.005**
*σ*|+*μ* strong		0.05	**0.005**

The simulated effects for the tested parameters are displayed in bold font.

Finally, beta distributed data were simulated as a parametric bootstrap sample [42]$y_{j}^{*}∼\text{BE}(\mu ={\hat{\mu }}_{j},\sigma ={\hat{\sigma }}_{j})$ for each CpG site. The resulting data were beta distributed with known effects of the covariate BMI on location and scale, in absence or presence of nuisance effects on the respective other parameter.

The eight competing models (Table 1) were fitted to these data, and observed type I error rate and power of the test with the null hypothesis *H*_0_:*γ*_BMI_=0 were estimated as the proportion of *p*-values below 0.05, given *γ*_BMI_=0 or *γ*_BMI_=0.005, respectively. The analysis was conducted at *n*=1763 and *n*=250 as before.

#### Real data setting

To investigate the influence of the response distribution on test performance, data were generated having the distribution of the real *β*-values, while covariate effects were maintained. This was achieved by reassigning the originally observed methylation values to the subjects, according to the ranks of the subjects’ simulated response values. Thereby, the simulated covariate effects were largely maintained, while the original response distribution was reconstructed. The eight competing models were fitted to these data, and observed type I error rates and power were estimated.

## Results and discussion

### Comparison of competing models for methylation data

**Figure 1:**
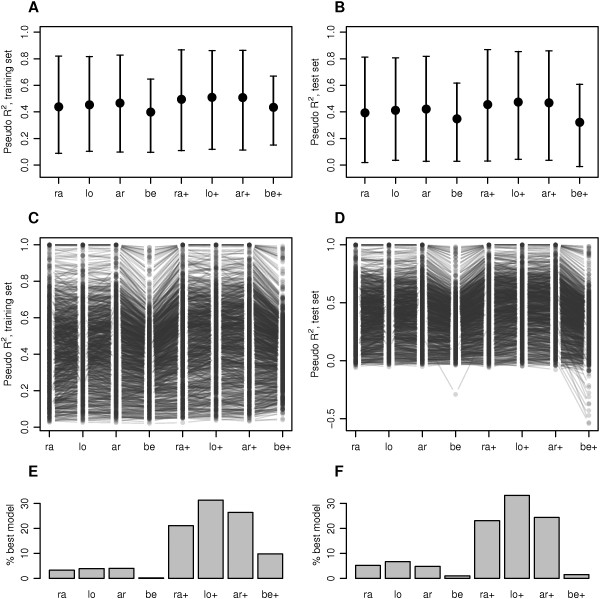
**Performance of competing models for DNA methylation data. A and B** Median, 5% and 95% quantile of pseudo R ^2^ in training and test data set, respectively, across the CpG sites. **C and D** Pseudo R ^2^ values of individual CpG sites in training and test data set, respectively. 1000 CpG sites were randomly chosen for this plot. **E and F** Proportion of CpG sites for which the respective model had the largest pseudo R ^2^ measure as compared to the competing models, in training and test data set, respectively. Model abbreviations are explained in Table 1.

Model fit and predictive performance were compared in terms of the pseudo R ^2^ criterion evaluated in training and test data, respectively, as described in detail above (total sample size *n*=1763). Among the fixed scale models, the beta regression model (be) performed worst on average (Figure 1A-D; median training R ^2^: ra 0.438, lo 0.454, ar 0.466, be 0.398; median test R ^2^: ra 0.393, lo 0.412, ar 0.422, be 0.348) and for the majority of CpG sites (Figure 1E and F) according to both criteria. For the Gaussian regression models, performance improved when also the scale parameter was modeled as a function of covariates, suggesting that effects of the modeled covariates on methylation scale are present. This was not as clearly observed for the beta regression model (be+), which performed considerably worse than the variable scale Gaussian regression models on raw (ra+) or transformed (lo+, ar+) data. Among the variable scale models, the Gaussian model on binary logit transformed data (lo+), i.e. on *M*-values, showed the best model fit (median training R ^2^: 0.510) and predictive performance (median test R ^2^: 0.474) on average (Figure 1A-D) and for the largest proportion (>30*%*) of CpG sites (Figure 1E and F).

**Figure 2:**
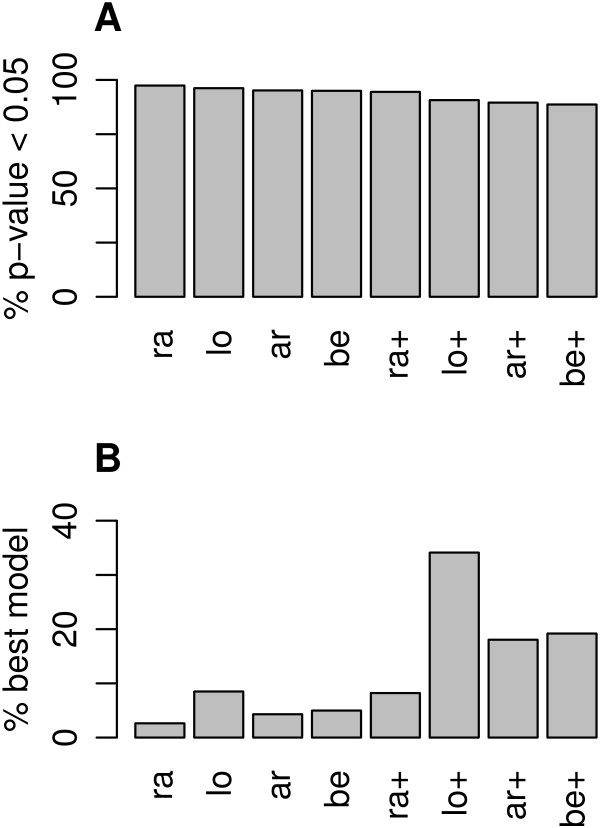
**Residual normal fit of competing models for DNA methylation data. A** Proportion of CpG sites for which significant deviation of residuals from normality was indicated by Shapiro-Wilk test *p*-value <0.05. **B** Proportion of CpG sites for which the respective model had the best residual normal fit as compared to the competing models. Model abbreviations are explained in Table 1.

When the analyses were repeated with a reduced sample size (*n*=250), training set R ^2^ values indicated a substantial increase in model fit for variable scale as compared to fixed scale models (Figure S2 in Additional file 3). At the same time, test set R ^2^ values were strongly deflated for a part of the CpG sites, indicating diminished predictive performance in the presence of overparametrization.Distribution assumption in the competing models was checked through residual normality tests. As shown in Figure 2A, a significant deviation from residual normality was indicated for more than 94% of CpG sites for all fixed scale models, and for more than 88% of CpG sites for all variable scale models. In contrast, parametric bootstrap draws from the fitted variable scale beta regression model showed a deviation from residual normality for 5% of the CpG sites in the be+ model, as expected (not shown). Thus, none of the competing models fitted well to more than 12% of CpG sites, suggesting that at the majority of CpG sites, methylation followed neither a beta distribution, nor a normal distribution after any of the investigated transformations.

The unexpectedly bad beta distribution fit might be attributed to an error component arising from the microarray experiment [43]. Of note, the two assay designs present on the Infinium HumanMethylation 450K BeadChip differed in the severity of the deviation from beta distribution fit.

When we compared the eight models in terms of severity of deviation from residual normality, model fit was improved when also the scale parameter was modeled as a function of covariates (Figure 2B). Overall, the variable scale Gaussian model on *M*-values (lo+) showed the best residual normality fit in the majority (34.12%) of CpG sites.

### Performance of hypothesis tests

Next, we investigated how strongly the violation of the distribution assumptions of the competing models, specifically the beta regression model, affected the performance of downstream t-tests for covariate effects. Specifically, we explored the impact of known covariate effects on one distribution parameter on the performance of tests for the effect of this covariate on the other distribution parameter. For this purpose, in a simulation study beta distributed and real-data distributed methylation responses were generated with known covariate effects on location and/or scale (see Methods section and Table 2).

#### Observed type I error rates in beta distributed data

**Figure 3:**
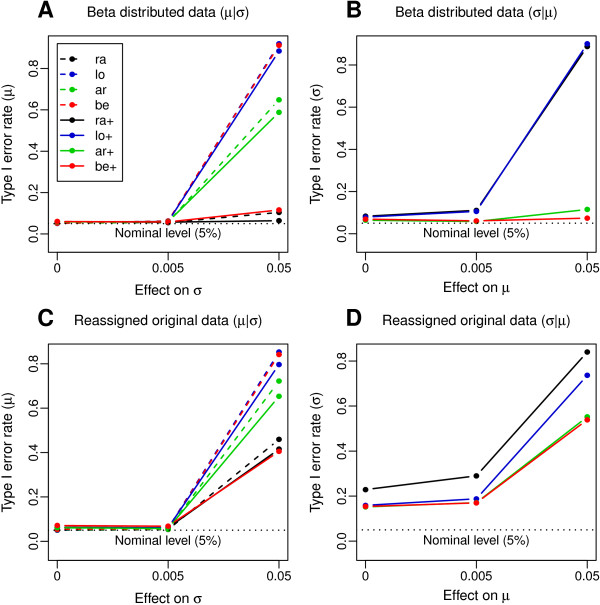
**Simulation study: Observed type I error rates of hypothesis tests for covariate effects.** Observed type I error is plotted against the effect size that the same covariate (BMI) had on the other distribution parameter. Simulation results are shown for beta distributed **(A, B)** and for real-data distributed methylation values **(C, D)**. Model abbreviations are explained in Table 1.

First, observed type I error rates of tests for covariate effects on the location parameter *μ* (*μ*|*σ* setting) were investigated in beta distributed data at full sample size (*n*=1763). Here, all models met the nominal level *α*=0.05 on average if no effect of the same covariate on the scale parameter *σ* was present (Figure 3A; Figure S3A, C and E in Additional file 3). In the presence of a weak covariate effect on *σ*, which was chosen to be within the range of actually observed covariate effects on the methylation data, all models adhered to their norminal level. When strong effects of the same covariate on *σ* were present, the models be, lo and lo+ (observed type I error rate >88%) and ar as well as ar+ (observed type I error rate >58%) showed severely inflated type I error rates. In contrast, the models be+ and ra showed moderately inflated type I error rates (be+: 11.6%, ra: 10.4%), whereas the nominal level was met in the case of the model ra+.

Next, we investigated tests for covariate effects on the scale parameter *σ* in beta distributed data (*σ*|*μ* setting). The models be+ and ar+ almost adhered to their nominal level irrespective of covariate effects on *μ*, whereas ra+ and lo+ showed a mild inflation of type I error rates (>10%) in the presence of weak effects on *μ*, which increased to above 88% when the covariate effect on *μ* was strong (Figure 3B; Figure S3B, D and F in Additional file 3).

Exemplarily, we provide theoretical considerations explaining the increased type I error rates observed for the different models in the *σ*|*μ* setting. Since the simulated data were beta distributed, their variance depended on the mean, with *Var*(*y*)=*μ*(1-*μ*)*σ*^2^ (compare Methods section). In the variable scale Gaussian model on raw data (ra+), the parameter

$${\tilde{\sigma }}_{i}=\sqrt{\text{Var}\left(y_{i}\right)}=\sqrt{\mu_{i}\left(1-\mu_{i}\right)\sigma^{2}}=\sigma \sqrt{\mu_{i}\left(1-\mu_{i}\right)}$$

is modeled. In the setting where covariate effects on *μ* are present but not on *σ*, ${\tilde{\sigma }}_{i}$ would still depend on covariates through *μ*_*i*_, so that effects on *μ* might be falsely attributed to *σ*, explaining the increased type I error rate. Similarly, in the Gaussian model on binary logit transformed data (lo+), applying the delta method yields

$$\begin{array}{l} \begin{array}{cc} {\tilde{\sigma }}_{i} & =\sqrt{\text{Var}\left(n2\left(\frac{y_{i}}{1-y_{i}}\right)\right)}\\ \approx \sqrt{\text{Var}\left(y_{i}\right)}\cdot \frac{1}{\text{log}(2)\mu_{i}\left(1-\mu_{i}\right)}=\sigma \cdot \frac{1}{\text{log}(2)\sqrt{\mu_{i}\left(1-\mu_{i}\right)}} \end{array} \end{array}$$

which depends on *μ*_*i*_ as well. Again, this explains an increased type I error rate for *σ*. In the special case of arcsine square root transformed data,

$$\begin{array}{lll} {\tilde{\sigma }}_{i} & =\sqrt{\text{Var}\left(\text{si}n^{-1}\left(\sqrt{y_{i}}\right)\right)}\; & \;\\ & \approx \sqrt{\text{Var}\left(y_{i}\right)}\cdot \sqrt{\frac{1}{2\mu_{i}\left(1-\mu_{i}\right)}}=\sigma \cdot \frac{1}{\sqrt{2}}.\; & \; \end{array}$$

Here, ${\tilde{\sigma }}_{i}$ is approximately independent of *μ*_*i*_, explaining the good performance of the ar+ model in the *σ*|*μ* setting.

#### Observed type I error rates in the real-data scenario

Next, analyses were repeated with real-data distributed methylation responses, which had exactly the same marginal distributions as the original data. In contrast to beta distributed data, none of the models adhered to the nominal level in the presence of strong covariate effects on the other distribution parameter (Figure 3C and D; Figure S4 in Additional file 3), with type I errors of above 40% observed for all models in both *μ*|*σ* and *σ*|*μ* settings.

Although methylation data are bounded in the unit interval with a dependency between mean and variance, they show deviation from the beta distribution (Figure 2) so that the relation between mean and variance might not be correctly reflected by *Var*(*y*)=*μ*(1-*μ*)*σ*^2^. Consequently, effects on either location or scale parameter might be falsely attributed to the other during model fitting.

In addition, in the *σ*|*μ* setting, type I error rates larger than 15% were observed for all models, even in absence of covariate effects on *μ*. They were largest (22.9%) for the ra+ model (Figure 3D). This suggests that tests on the scale parameter are more sensitive towards violations of the distribution assumption than tests on the location parameter.

**Figure 4:**
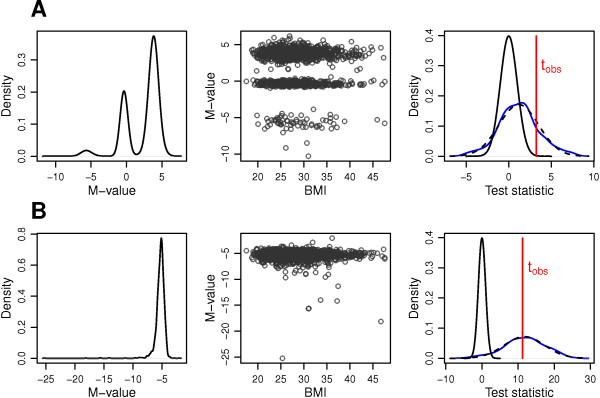
**Origins of inflated type I error rates of downstream hypothesis tests.** Two examples of CpG sites with missing covariates **(A)** and strong outlier structure **(B)**: From left to right - kernel density plot of methylation *M*-value, scatter plot of methylation *M*-value against BMI and kernel density plot of the test statistic null distribution assumed by the model lo+ (solid black line) and realized in the bootstrap samples after inclusion of genetic variants (solid blue line), for the test for a BMI effect on the scale parameter. The realized distribution approximately followed a normal distribution (dashed black line). *t*_*obs*_ and solid red line: test statistic from the original data without inclusion of genetic variants.

In our simulated data, two reasons seemed to be mainly responsible for this observation: missing covariates such as SNPs (Figure 4A), and outliers in the data (Figure 4B). It is not uncommon that SNPs located in close proximity to CpG sites strongly affect their methylation level [44], at some CpG sites resulting in multimodal marginal distributions of methylation values (as in Figure 4A). When these effects are not modeled and genotypes are unevenly distributed among the subjects with low and high covariate values - by chance or through a true SNP effect on the covariate - a falsely significant effect of the covariate on methylation scale is sometimes observed. Also, the fact that the investigated data were derived from whole blood might contribute to the increased type I error rates. Whole blood represents a mixture of different blood cells, which are known to show cell-specific methylation patterns [19]. The applied method to estimate and then adjust for selected white blood cell proportions might not fully remove the mixture effect from the methylation data. Furthermore, tests for covariate effects on *σ* are specifically sensitive towards outliers in the methylation data (Figure 4B). A potential solution to these problems is presented in the next section.

When the analyses were repeated in a smaller sample (*n*=250), an additional trend emerged: observed type I error was systematically larger for variable scale than for fixed scale models even in absence of covariate effects on the other distribution parameter (Figure S5 in Additional file 3). This was observed for both beta distributed and real-data distributed methylation responses, and was in line with the reduced predictive performance of variable scale models in the smaller sample (Figure S2 in Additional file 3). Together these findings suggest overfitting.

#### Observed power

Finally, we compared the models in terms of their power to detect existing covariate effects of moderate strength. We observed that power of downstream t-tests was generally smaller to detect similarly sized covariate effects on *σ* than on *μ* (Figure S6 in Additional file 3). However, none of the models clearly outperformed the others in terms of power in absence of covariate effects on the other distribution parameter. The presence of weak effects on *σ* tended to reduce power to detect effects on *μ* for all models. When strong effects on *σ* were simulated, type I error superimposed power, so that results are difficult to interpret (Figure S6 in Additional file 3).

### Resampling procedure for models for location and scale

Since asymptotic inference for variable scale models for methylation data is associated with inflated type I errors, we next considered resampling-based modes of inference. Standard parametric bootstrap is not an option for DNA methylation data, since we do not know the true distribution of the data. In non-parametric bootstrap, i.e. sampling of observations with replacement, the problem arises that the mean-variance structure within each observation is maintained, and false positives due to identifiability problems between covariate effects on the two distribution parameters would not be corrected for. This problem might occur in a reverse manner in permutation testing. Therefore, we developed a resampling procedure for models for location and scale (Algorithm 1), which provides a solution for both the unknown distribution of methylation data and false positives arising from covariate effects on the respective other distribution parameter.

**Figure 5:**
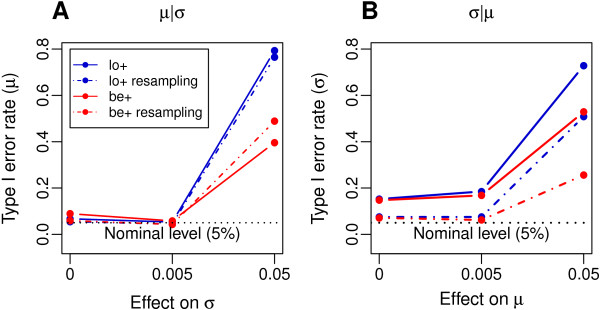
**Type I error control through the resampling procedure and inclusion of genetic variants as covariates.** Observed type I error for **(A)***μ* and **(B)***σ* is plotted against effect size that the same covariate (BMI) had on the other distribution parameter. Simulation on real-data distributed methylation responses, before (solid lines) and after (dotdashed lines) application of the resampling procedure and inclusion of genetic variants as covariates. Model abbreviations are explained in Table 1.

To evaluate the performance of the developed resampling procedure, we applied it to the real-data distributed methylation values generated in the simulation study for 10,000 CpG sites (see Methods section), for the variable scale models lo+ and be+, with *B*=100. In addition, we identified for each CpG site the three SNPs from a ±5 Mb window around the CpG site showing successively the strongest correlation with methylation at this site [4]. These SNPs were then included as additional covariates in the model. For both lo+ and be+ models, this combined strategy substantially reduced type I error rate close to the nominal level, as long as covariate effects on the other distribution parameter were moderate (Figure 5). 

### Validation in a data set of acute lymphoblastic leukemia patients and healthy controls

An important question is whether our observations are valid also for methylation data from cancer patients and healthy controls, which might differ fundamentally from population-based data in terms of their distribution and outlier structure. These aspects might have an impact on the performance of the different models, as well as on the performance of the proposed resampling strategy. Therefore, we repeated all performance comparisons and evaluations on a publicly available data set comprising Infinium HumanMethylation 450K methylation data from bone marrow samples of 615 acute lymphoblastic leukemia (ALL) patients of two different types (B-cell precursor ALL and T-cell ALL), as well as 80 healthy controls (GEO accession number: GSE49031 [45]; see Additional file 1 for methodological details).

In these data, we were able to confirm our main findings: First, models of location and scale outperformed models of location only in terms of model fit and predictive performance (Figure S7 in Additional file 3). Second, the Gaussian variable scale model on methylation *M*-values most often showed the best residual model fit (Figure S8 in Additional file 3). Third, we again observed increased type I error rates for tests of covariate effects on the scale parameter, and on both distribution parameters in the presence of strong effects on the respective other parameter (Figure S9 in Additional file 3). Finally, although genetic confounding could not be accounted for due to lack of SNP data, the proposed resampling precedure performed considerably well in the moderate effect size scenario (Figure S10 in Additional file 3).

It remains a challenge to improve the method to control type I error in the presence of strong covariate effects on the other distribution parameter. In the population-based KORA data, effects of lifestyle and phenotypic factors were rarely stronger than the simulated moderate effect size. In contrast, in the investigated cancer data set, case-control effects were sometimes larger than the simulated moderate effect size, so that the proposed resampling procedure will not be capable of clearly separating effects on location and scale. A similar issue might arise when investigating the effect of proportions of white blood cell types, which tend to have strong effects on specific CpG sites, affecting both mean [39] and variability [27]. However, we would like to emphasize that a clear separation of covariate effects on location and scale in the presence of a strong (but unknown) relation between the two is a major challenge to any parametric or nonparametric approaches.

### Application to an EWAS of BMI and age in the KORA study

Our results have shown that it is worth to examine covariate effects on DNA methylation variability, since model performance was improved by modeling the scale parameter. Therefore, we undertook an EWAS of BMI and age in the large population-based KORA F4 sample. We used the Gaussian model on *M*-values (lo+) which achieved the best model fit and predictive performance in our model comparisons. BMI was chosen since obesity has been reported before to associate with DNA methylation variability at specific CpG sites [9]. In addition, we were interested in age since a recent investigation has revealed the presence of regions in the genome that are characterized by an increased methylation variability in an age group specific manner, where significantly more of these regions were observed in older age groups [26].

To avoid inflated type I errors of tests for covariate effects on location and scale, we included SNPs as additional covariates as described above. Otherwise, all covariates were included that were also included in the model comparisons. In addition, we used the resampling procedure described above for the significant associations.

**Figure 6:**
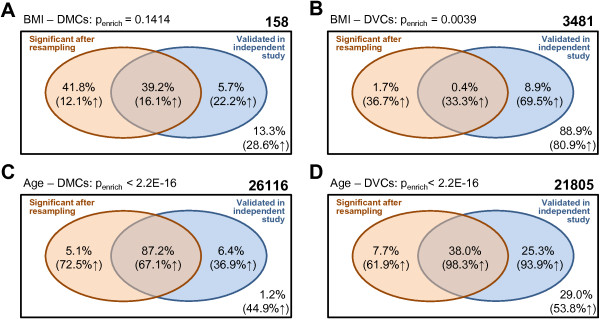
**EWAS results.** Results for BMI **(A and B)** and age **(C and D)** effects on methylation level (*μ*) and variability (*σ*) are shown, respectively. Bold number in the right top corner: Number of CpG sites with genome-wide significant association (*p*<1.3·10^-7^) according to asymptotic test results. Numbers in the box represent percentages of associations that were significant according to resampling-based inference (red circle) and/or that were validated in the independent F3 study (at *p*<0.05, blue circle), or neither of them (bottom right). Numbers in brackets indicate the respective percentage of positive associations. *p*-values are from Fisher’s exact tests for enrichment of validated associations among the resampling-significant associations.

Before the resampling procedure was applied, 158 and 3481 genome-wide significant associations between BMI and methylation level (*μ*) as well as variability (*σ*) were observed, respectively (*p*<1.3·10^-7^, corresponding to Bonferroni correction for multiple testing) (Figure 6A and B). We term these CpG sites differentially methylated CpG sites (DMCs) and differentially variable CpG sites (DVC), respectively, in accordance with Xu *et al.*[9]. Larger numbers of significant associations (26116 DMCs and 21805 DVCs) were observed for age (Figure 6C and D). As expected from the test performance results, the percentage of CpG sites for which significance was “confirmed” using the resampling procedure was larger for DMCs (BMI: 80.1%, age: 92.3%) than for DVCs (BMI: 2.1%, age: 45.7%). Specifically in the case of BMI, the majority of DVCs seemed to be false positives according to the resampling procedure.

For BMI DVCs, age DMCs and age DVCs, we observed that associations with significant resampling-based *p*-values could be validated significantly more often in the independent KORA F3 study (Figure 6B-D). This observation was stable to the threshold of significance in the replication study. For instance, when the threshold was changed from nominal significance (*p*≤0.05) to a Bonferroni-corrected threshold (*p*≤0.05/{number of tests}) which is often used, enrichment results essentially did not change. Assuming that validation in an independent study is an indicator of trueness of an effect (ignoring the presence of unaccounted confounders), these results suggest that the resampling procedure filtered out false positives. Note that the replication study was smaller (*n*=486) than the discovery study (*n*=1763), so that not all true effects can be expected to validate.

Investigating the observed effect directions, we noted that for the majority (79.9%) of BMI DVCs that were not confirmed by resampling, associations were positive (*p*=4.9·10^-16^). This was not observed for the DVCs significant after resampling. A plausible explanation might be that the variable scale model is more susceptible towards methylation outliers at larger BMI values where BMI density is sparser. Interestingly, Xu *et al.*[9] reported an enrichment of positive associations of obesity and methylation variability in a study population of 48 obese and 48 lean subjects, where the standard deviation of BMI in the obese group was fivefold that of the lean group. The identified DVCs were often characterized by one or several outliers occuring in the obese group. The authors used the parametric Bartlett’s test to identify DVCs. To evaluate this test procedure using our own data, we subjected the 3481 initially discovered BMI DVCs to Bartlett’s test after dividing the study population into two groups by the median BMI. Although this approach differs considerably from ours, for instance since Bartlett’s test does not include covariate information, and the variability is modeled separately from the mean, Bartlett’s test indicated genome-wide significance for 1893 (54.4%) of the DVC initially identified by GAMLSS, 80.3% of which were positive associations. This suggests that Bartlett’s test might share the susceptibility to outlier values and likely also to genetic confounding in the methylation data, and might have increased type I error rates in that case.

In the EWAS of age, a substantial number of DMCs (22774, 87.2%) and DVCs (8279, 38%) passed both resampling-based correction and validation step (Figure 6C and D). The vast majority of validated DVCs (98.3%) showed positive associations (*p*-values <2.2·10^-16^ as compared to unvalidated CpG sites), indicating increased methylation variability with increasing age. This observation fits well with the previous finding of larger DNA methylation variability in adult as compared to newborn blood [27]. It is also in agreement with an increased number of age group specific highly variable CpG sites in older as compared to younger age groups [26]. Specifically, we also observed DVCs at neurotransmission-related genes. In addition, highly variable CpG sites were enriched in the vicinity of genes involved in developmental and morphogenetic processes [46,47], suggesting a role of methylation variability during development. Age-related increases in methylation variability at specific CpG sites might be attributed to both stochastic events [46] as well as accumulating environmental and lifestyle influences. We also have to acknowledge the possibility that the observed age effects on methylation variability are partially attributed to changes in white blood cell proportions with age [48]. White blood cell types differ strongly in methylation variability [27]. Although we included estimated white blood cell proportions as covariates, residual confounding might occur [48].

## Conclusions

We have addressed two challenges arising from the characteristics of methylation data: First, the appropriate treatment of methylation *β*-values as a proportion response, and second, the difficulty of assessing covariate effects on both location (mean) and scale (variability) of these data. The latter issue has become important because recent findings suggest a role of methylation variability in addition to methylation level in disease processes, including cancer [21-25]. In contrast to recent strategies to assess associations between methylation variability and disease-related traits [9,25-27], we propose *simultaneous* modeling of location and scale using the GAMLSS framework [28].

A key result of our study is that simultaneous modeling of mean and variability of DNA methylation data improved the predictive performance as compared to modeling the mean only. A particularly good performance was observed for the Gaussian model on methylation *M*-values. To avoid false positives arising from violations of the distribution assumption, we proposed and applied a resampling procedure as a mode of inference for models for location and scale. In our experiments, this procedure substantially reduced type I error rates so that they became close to the nominal level in practically relevant settings. The validity of this approach could be confirmed both in population-based data and in a data set of cancer patients and healthy controls. Moreover, the application of our methodology to an EWAS of BMI and age in the large population-based KORA F4 study revealed biologically plausible positive effects of age on methylation variability. These effects were stable towards validation in an independent study.

Our findings suggest that GAMLSS is a useful tool to explore environmental and lifestyle effects on methylation variability, simultaneously to effects on the mean. Since the investigated models for location and scale were susceptible towards overfitting when sample size was moderate, it could be promising to investigate extensions based on regularization techniques such as boosting (which has been implemented for GAMLSS [49]). In addition, in our data, methylation at the majority of CpG sites follows none of the compared distributions. Thus, it might be relevant to explore robust methods such as quantile regression [50].

## Competing interests

The authors declare that they have no competing interests.

## Authors’ contributions

SW, NF and MS devised the basic idea for the manuscript. SZ performed the laboratory measurements. SW performed the statistical analyses with contributions from NF and MS. KS, CG, MW and HG contributed data and obtained funding. SW, NF, SZ, KS, CG, MW, HG and MS wrote the manuscript. All authors read and approved the final manuscript.

## Supplementary Material

Additional file 1 **Supplementary methods.** DNA methylation data preprocessing; Definition of the pseudo R ^2^ criterion for the competing models. Data preprocessing and methods for the ALL data set. Click here for file

Additional file 2 **Supplementary tables.Table S1.** Description of the KORA F4 study population. **Table S2.** Description of the KORA F3 study population. Click here for file

Additional file 3 **Supplementary figures.Figure S1.** Distribution of *β*-, *M*- and *A*-values. **Figure S2.** Performance of competing models for DNA methylation data (n = 250). **Figure S3.** Simulation study: Distribution of type I error rates of hypothesis tests for covariate effects in beta distributed methylation responses (n = 1763). **Figure S4.** Simulation study: Distribution of type I error rates of hypothesis tests for covariate effects in real-data distributed methylation responses (n = 1763). **Figure S5.** Simulation study: Average estimated type I error rates of hypothesis tests for covariate effects (n = 250). **Figure S6.** Simulation study: Average estimated power of hypothesis tests for covariate effects (n = 1763). **Figure S7.** Performance of competing models for DNA methylation data in a data set of acute lymphoblastic leukemia (ALL) patients and healthy controls (n = 695). **Figure S8.** Residual normal fit of competing models for DNA methylation data in a data set of acute lymphoblastic leukemia (ALL) patients and healthy controls (n = 695). **Figure S9.** Simulation study: Average estimated type I error rates of hypothesis tests for covariate effects (ALL data set; n = 695). **Figure S10.** Type I error control through the resampling procedure in a data set of acute lymphoblastic leukemia (ALL) patients and healthy controls (n = 695). Click here for file
