# Supplementary material for: On the potential of models for location and scale for genome-wide DNA methylation data
Source: BMC Bioinformatics. 2014 Jul 3;15:232. doi: 10.1186/1471-2105-15-232 (PMC4227139; doi:10.1186/1471-2105-15-232)
Supplement: Additional file 1 — Supplementary methods. DNA methylation data preprocessing; Definition of the pseudo R 2 criterion for the competing models. Data preprocessing and methods for the ALL data set. [file 1471-2105-15-232-S1.pdf]

On the potential of models for location and scale for genome-wide DNA methylation data

Simone Wahl, Nora Fenske, Sonja Zeilinger, Karsten Suhre, Christian Gieger, Melanie Waldenberger, Harald Grallert, Matthias Schmid

Additional file 1: Supplementary Methods

DNA methylation data preprocessing in the KORA data set

Raw methylation data were extracted with Illumina GenomeStudio Version 2011.1, Methylation Module 1.9.0 and preprocessed using R, version 3.0.1 [1]. Some preprocessing steps were adopted from the pipeline proposed by Touleimat and Tost [2]. First, probes with signals being summarized from less than three functional beads, and probes associated with a detection p-value larger than 0.01 were defined as low-confidence probes. Samples with more than 20% low-confidence probes were removed from the data set. Second, sites representing or being located in 50 bp proximity to single nucleotide polymorphisms (SNPs) with a minor allele frequency of at least 5% were excluded from the data set to avoid confounding of the methylation level by genetic variation. Third, color bias adjustment using smooth quantile normalization, and background correction based on the negative control probes present on the BeadChip, separately for the two color channels, were conducted on the β-values using the R package `lumi`, version 2.12.0 [3].

In addition, β-values corresponding to low-confidence probes were set to missing, and CpG sites were subjected to a 95% callrate threshold, were CpG sites with more than 5% low-confidence probes were removed from the analysis. Then, beta-mixture quantile normalization (BMIQ) was applied to correct the shift in the distribution of the beta values of the Infinium I and II probes [4] using the R package `wateRmelon`, version 1.0.3 [5]. Finally, to avoid ambiguous methylation signals derived from probes co-hybridizing to highly homologous genomic sequences other than the target sequence, such probes, as predicted by Chen *et al.* 2013 [6], were removed prior to analysis. X and Y chromosomes were removed from the epigenome-wide association study (EWAS) on age and BMI.

To avoid technical confounding of the investigated phenotype-methylation associations, we performed a principal component analysis on the positive control probes present on the BeadChip, assuming that differences in control probe levels reflect technical differences between samples (John Chambers, personal communication). The first 15 principal components (PCs) were then included as covariates in the location submodels in all analyses.

Definition of the pseudo R^2 criterion for the competing models

The pseudo R^2 criterion [7, 8] is defined as:

$$\mathrm{R}^2 = 1 - \left(\frac{L_0}{L_1}\right)^{2/n} = 1 - exp\left(-\frac{2}{n}\left(l_1 - l_0\right)\right),$$

where L_0 and L_1 represent the likelihoods of the intercept-only and the full model, respectively, while l_0 and l_1 represent the corresponding log-likelihoods.

Thereby, log-likelihoods were derived for the different models as follows: For the models with a transformed response $\tilde{y}$, the transformation theorem

$$f_Y(y) = f_{\tilde{Y}}\left(h^{-1}(y)\right)\left|\frac{dh^{-1}(y)}{dy}\right|$$

was applied, where for the logit2 transformation ($h^{-1}(y) = log_2\left(\frac{y}{1-y}\right)$):

$$\frac{dh^{-1}(y)}{dy} = \frac{1}{log(2)}\frac{1-y}{y}\frac{1}{(1-y)^2} = \frac{1}{log(2)y(1-y)}$$

and for the arcsine square root transformation ($h^{-1}(y) = arcsine\left(\sqrt{y}\right)$):

$$\frac{dh^{-1}(y)}{dy} = \frac{1}{\sqrt{1-\sqrt{y}^2}}\frac{1}{2\sqrt{y}} = \frac{1}{2\sqrt{y(1-y)}}.$$

Thus, the following log-likelihoods resulted for the different models, evaluated at the fitted distribution parameters $\hat{\mu}_i$ and $\hat{\sigma}_i$, $i = 1, \ldots, n$, of the fitted and intercept-only models, respectively:

- for Gaussian regression on the raw data (ra, ra+):

$$l(\boldsymbol{\mu}, \boldsymbol{\sigma}|\boldsymbol{y})\Big|_{\hat{\boldsymbol{\mu}},\hat{\boldsymbol{\sigma}}} = \sum_{i=1}^{n}\left(-\frac{1}{2}log(2\pi) - log(\hat{\sigma}_i) - \frac{(y_i - \hat{\mu}_i)^2}{2\hat{\sigma}_i^2}\right) \tag{1}$$

- for Gaussian regression on the logit2-transformed data (lo, lo+):

$$l(\boldsymbol{\mu}, \boldsymbol{\sigma}|\boldsymbol{y})\Big|_{\hat{\boldsymbol{\mu}},\hat{\boldsymbol{\sigma}}} = \sum_{i=1}^{n}\left(-\frac{1}{2}log(2\pi) - log(\hat{\sigma}_i) - \frac{\left(log_2(\frac{y_i}{1-y_i}) - \hat{\mu}_i\right)^2}{2\hat{\sigma}_i^2} - log\left(log(2) - log(y_i(1-y_i))\right)\right)$$

- for Gaussian regression on the arcsine square root-transformed data (ar, ar+):

$$l(\boldsymbol{\mu}, \boldsymbol{\sigma}|\boldsymbol{y})\Big|_{\hat{\boldsymbol{\mu}},\hat{\boldsymbol{\sigma}}} = \sum_{i=1}^{n}\left(-\frac{1}{2}log(2\pi) - log(\hat{\sigma}_i) - \frac{\left(arcsine(\sqrt{y_i}) - \hat{\mu}_i\right)^2}{2\hat{\sigma}_i^2} - log\left(2\sqrt{y_i(1-y_i)}\right)\right)$$

- and for beta regression on the raw data (be, be+):

$$l(\boldsymbol{\mu}, \boldsymbol{\sigma}|\boldsymbol{y})\Big|_{\hat{\boldsymbol{\mu}},\hat{\boldsymbol{\sigma}}} = \sum_{i=1}^{n} log\left(\frac{\Gamma\left(\frac{1}{\hat{\sigma}_i^2} - 1\right)}{\Gamma\left((\frac{1}{\hat{\sigma}_i^2} - 1)\hat{\mu}_i\right)\Gamma\left((\frac{1}{\hat{\sigma}_i^2} - 1)(1-\hat{\mu}_i)\right)}\right)$$
$$+ \left((\frac{1}{\hat{\sigma}_i^2} - 1)\hat{\mu}_i - 1\right)log(y_i) + \left((\frac{1}{\hat{\sigma}_i^2} - 1)(1-\hat{\mu}_i) - 1\right)log(1-y_i).$$

Data preprocessing and methods for the ALL data set

Methylation data from bone marrow samples of 615 acute lymphoblastic leukemia (ALL) patients (535 B-cell precursor type and 80 T-cell type) and 80 healthy controls were obtained from GEO (accession number: GSE49031; `http://www.ncbi.nlm.nih.gov/geo/query/acc.cgi?acc=GSE49031`). Data preprocessing has been described in detail [9]. Briefly, methylation levels were determined with the Infinium HumanMethylation 450K BeadChip, and methylation β-values were normalized using peak-based correction [10]. No batch effects were observed. Sites on the X and Y chromosomes and sites with genetic variation in the probes were removed, leaving data for 435,941 CpG sites for analysis [9].

All procedures described for the KORA data set were repeated on the ALL data set. For model comparison, in all location and scale submodels, the respective parameter was specified as a linear function of cancer status. Thereby, cancer status was defined as two dummy variables specifying T-ALL and BCL-ALL types. The additional inclusion of cancer subtypes did not influence observations (not shown). The resampling procedure was evaluated on the example of the effect of T-ALL status (as compared to healthy) on methylation.

References

[1] R Core Team: R: a Language and Environment for Statistical Computing. R Foundation for Statistical Computing, (2013). R Foundation for Statistical Computing

[2] Touleimat, N., Tost, J.: Complete pipeline for infinium(®) human methylation 450k beadchip data processing using subset quantile normalization for accurate dna methylation estimation. Epigenomics **4**(3), 325–341 (2012)

[3] Du, P., Kibbe, W.A., Lin, S.M.: lumi: a pipeline for processing illumina microarray. Bioinformatics **24**(13), 1547–1548 (2008)

[4] Teschendorff, A.E., Marabita, F., Lechner, M., Bartlett, T., Tegner, J., Gomez-Cabrero, D., Beck, S.: A beta-mixture quantile normalization method for correcting probe design bias in illumina infinium 450 k dna methylation data. Bioinformatics **29**(2), 189–196 (2013)

[5] Pidsley, R., Y Wong, C.C., Volta, M., Lunnon, K., Mill, J., Schalkwyk, L.C.: A data-driven approach to preprocessing illumina 450k methylation array data. BMC Genomics **14**, 293 (2013)

[6] Chen, Y.-a., Lemire, M., Choufani, S., Butcher, D.T., Grafodatskaya, D., Zanke, B.W., Gallinger, S., Hudson, T.J., Weksberg, R.: Discovery of cross-reactive probes and polymorphic cpgs in the illumina infinium humanmethylation450 microarray. Epigenetics **8**(2), 203–209 (2013)

[7] Maddala, G.S.: Limited-Dependent and Qualitative Variables in Econometrics. Cambridge University Press, Cambridge (1983)

[8] Cox, D.R., Snell, J.E.: Analysis of Binary Data. Chapman & Hall, London (1989)

[9] Nordlund, J., Bäcklin, C.L., Wahlberg, P., Busche, S., Berglund, E.C., Eloranta, M.-L., Flaegstad, T., Forestier, E., Frost, B.-M., Harila-Saari, A., Heyman, M., Jónsson, O.G., Larsson, R., Palle, J., Rönnblom, L., Schmiegelow, K., Sinnett, D., Söderhäll, S., Pastinen, T., Gustafsson, M.G., Lönnerholm, G., Syvänen, A.-C.: Genome-wide signatures of differential dna methylation in pediatric acute lymphoblastic leukemia. Genome Biol **14**(9), 105 (2013). doi:10.1186/gb-2013-14-9-r105

[10] Dedeurwaerder, S., Defrance, M., Calonne, E., Denis, H., Sotiriou, C., Fuks, F.: Evaluation of the infinium methylation 450k technology. Epigenomics **3**(6), 771–784 (2011)
