# Supplementary material for: On the potential of models for location and scale for genome-wide DNA methylation data
Source: BMC Bioinformatics. 2014 Jul 3;15:232. doi: 10.1186/1471-2105-15-232 (PMC4227139; doi:10.1186/1471-2105-15-232)
Supplement: Additional file 2 — Supplementary tables.Table S1. Description of the KORA F4 study population. Table S2. Description of the KORA F3 study population. [file 1471-2105-15-232-S2.pdf]

On the potential of models for location and scale for genome-wide DNA methylation data

Simone Wahl, Nora Fenske, Sonja Zeilinger, Karsten Suhre, Christian Gieger, Melanie Waldenberger, Harald Grallert, Matthias Schmid

Additional file 2: Supplementary Tables

Table S1: **Description of the KORA F4 study population.** BMI, body mass index; WBC, white blood cell count.

Variable	Mean (sd)	Median (range)
Age (years)	60.9 (8.9)	61.0 (32.0, 81.0)
BMI (kg/m2)	28.1 (4.7)	27.5 (17.5, 47.6)
WBC (/nl)	5.9 (1.6)	5.6 (2.7, 14.0)
Alcohol intake (g/d)	15.2 (19.5)	7.5 (0.0, 114.3)
	**Absolute frequencies**	**Relative frequencies (%)**
Sex (male/female)	857/906	48.6/51.4
Smoking state (never/former/current smoker)	779/728/256	44.2/41.3/14.5
Physical activity (active/inactive)	1012/751	57.4/42.6

Table S2: **Description of the KORA F3 study population.** BMI, body mass index; WBC, white blood cell count.

Variable	Mean (sd)	Median (range)
Age (years)	53.0 (9.6)	53.0 (35.0, 82.0)
BMI (kg/m2)	27.2 (4.6)	26.7 (15.1, 45.3)
WBC (/nl)	7.3 (1.9)	7.1 (3.4, 15.1)
Alcohol intake (g/d)	16.1 (19.6)	7.7 (0.0, 106.0)
	**Absolute frequencies**	**Relative frequencies (%)**
Sex (male/female)	252/234	51.9/48.1
Smoking state (never/former/current smoker)	249/0/250	49.9/0/50.1
Physical activity (active/inactive)	241/245	49.6/50.4
