# Supplementary material for: On the potential of models for location and scale for genome-wide DNA methylation data
Source: BMC Bioinformatics. 2014 Jul 3;15:232. doi: 10.1186/1471-2105-15-232 (PMC4227139; doi:10.1186/1471-2105-15-232)
Supplement: Additional file 3 — Supplementary figures.Figure S1. Distribution of β-, M- and A-values. Figure S2. Performance of competing models for DNA methylation data (n = 250). Figure S3. Simulation study: Distribution of type I error rates of hypothesis tests for covariate effects in beta distributed methylation responses (n = 1763). Figure S4. Simulation study: Distribution of type I error rates of hypothesis tests for covariate effects in real-data distributed methylation responses (n = 1763). Figure S5. Simulation study: Average estimated type I error rates of hypothesis tests for covariate effects (n = 250). Figure S6. Simulation study: Average estimated power of hypothesis tests for covariate effects (n = 1763). Figure S7. Performance of competing models for DNA methylation data in a data set of acute lymphoblastic leukemia (ALL) patients and healthy controls (n = 695). Figure S8. Residual normal fit of competing models for DNA methylation data in a data set of acute lymphoblastic leukemia (ALL) patients and healthy controls (n = 695). Figure S9. Simulation study: Average estimated type I error rates of hypothesis tests for covariate effects (ALL data set; n = 695). Figure S10. Type I error control through the resampling procedure in a data set of acute lymphoblastic leukemia (ALL) patients and healthy controls (n = 695). [file 1471-2105-15-232-S3.pdf]

On the potential of models for location and scale for genome-wide DNA methylation data

Simone Wahl, Nora Fenske, Sonja Zeilinger, Karsten Suhre, Christian Gieger, Melanie Waldenberger, Harald Grallert, Matthias Schmid

Additional file 3: Supplementary Figures

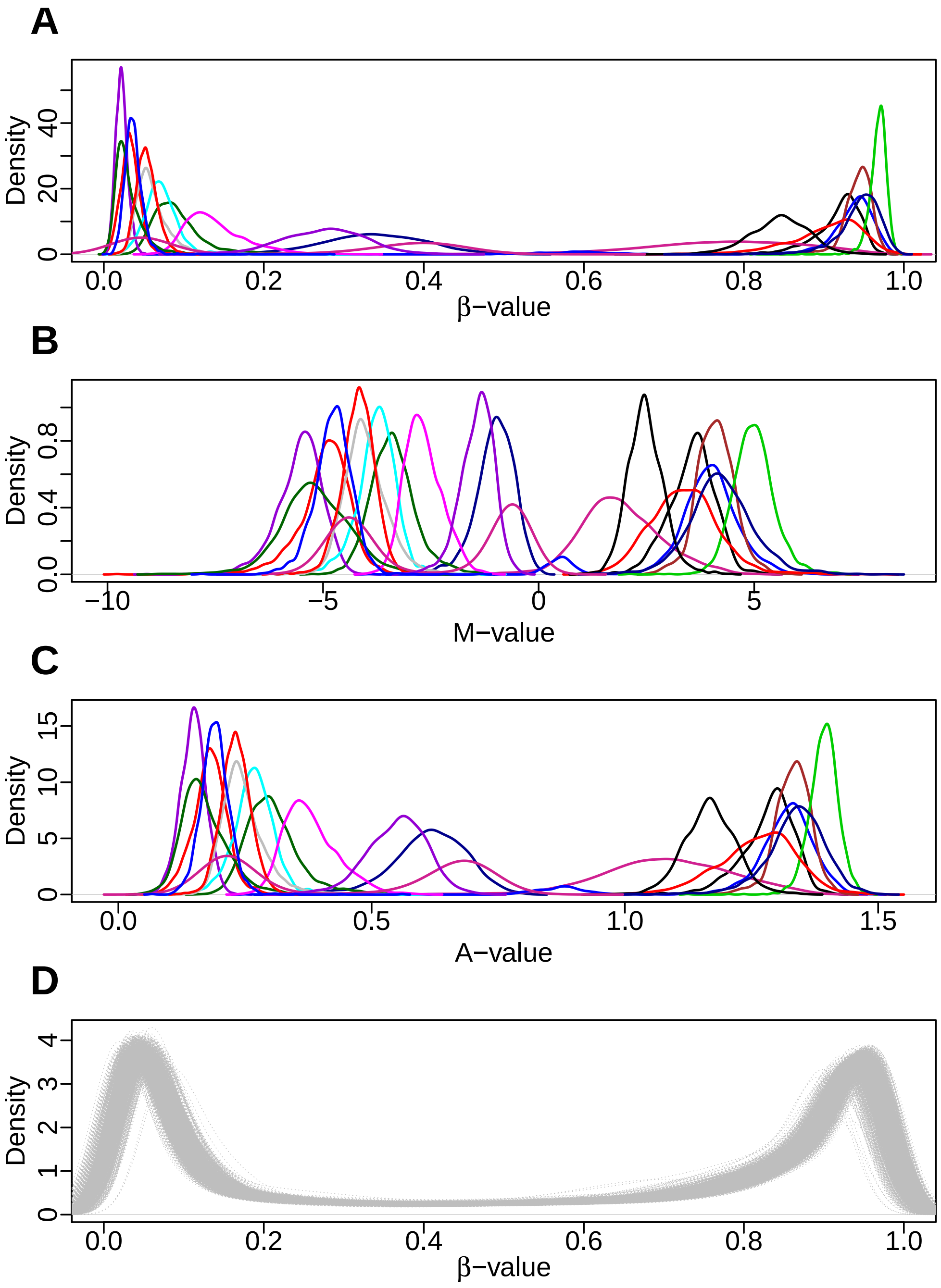

Figure S1: **Distribution of β-, M- and A-values.** **A, B and C** Kernel density plots of methylation β-values (A), and the corresponding M-values (B) and A-values (C) for 20 random CpG sites across the KORA F4 study population. **D** Kernel density plots of methylation β-values for each observation across all CpG sites in the data set. The majority of CpG sites are centered at a low (mode at a β-value of 0.045) or a high (mode at a β-value of 0.943) methylation state.

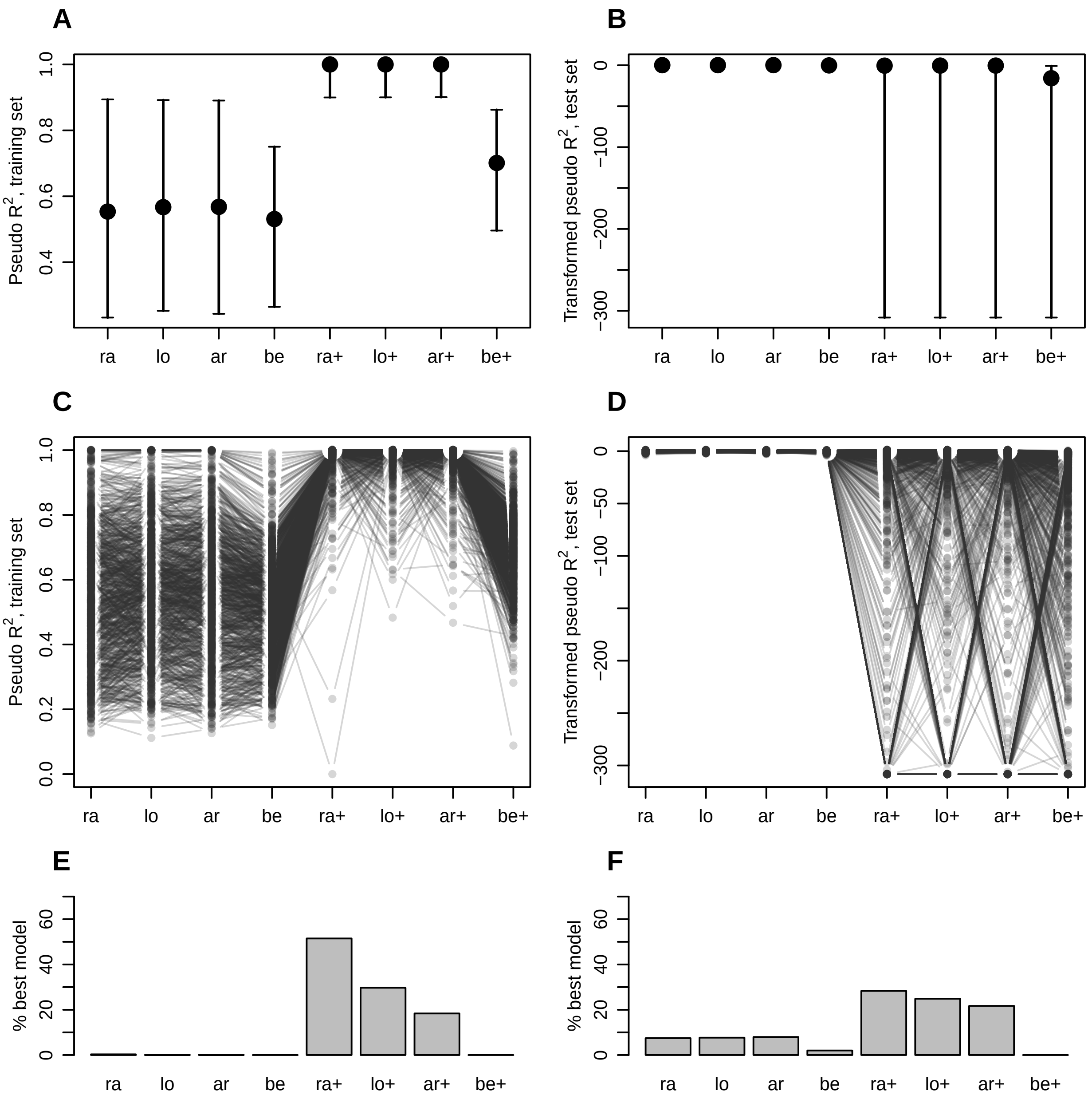

Figure S2: **Performance of competing models for DNA methylation data (KORA data; n = 250). A and B** Median, 5% and 95% quantile of pseudo R^2 in training and test data set, respectively, across the random set of the investigated CpG sites. **C and D** Pseudo R^2 values of individual CpG sites in training and test data set, respectively. 1000 CpG sites were randomly chosen for this plot. **D and E** Proportion of CpG sites for which the respective model had the largest pseudo R^2 measure as compared to the competing models, in training and test data set, respectively. Model abbreviations are explained in Table 1 in the main text.

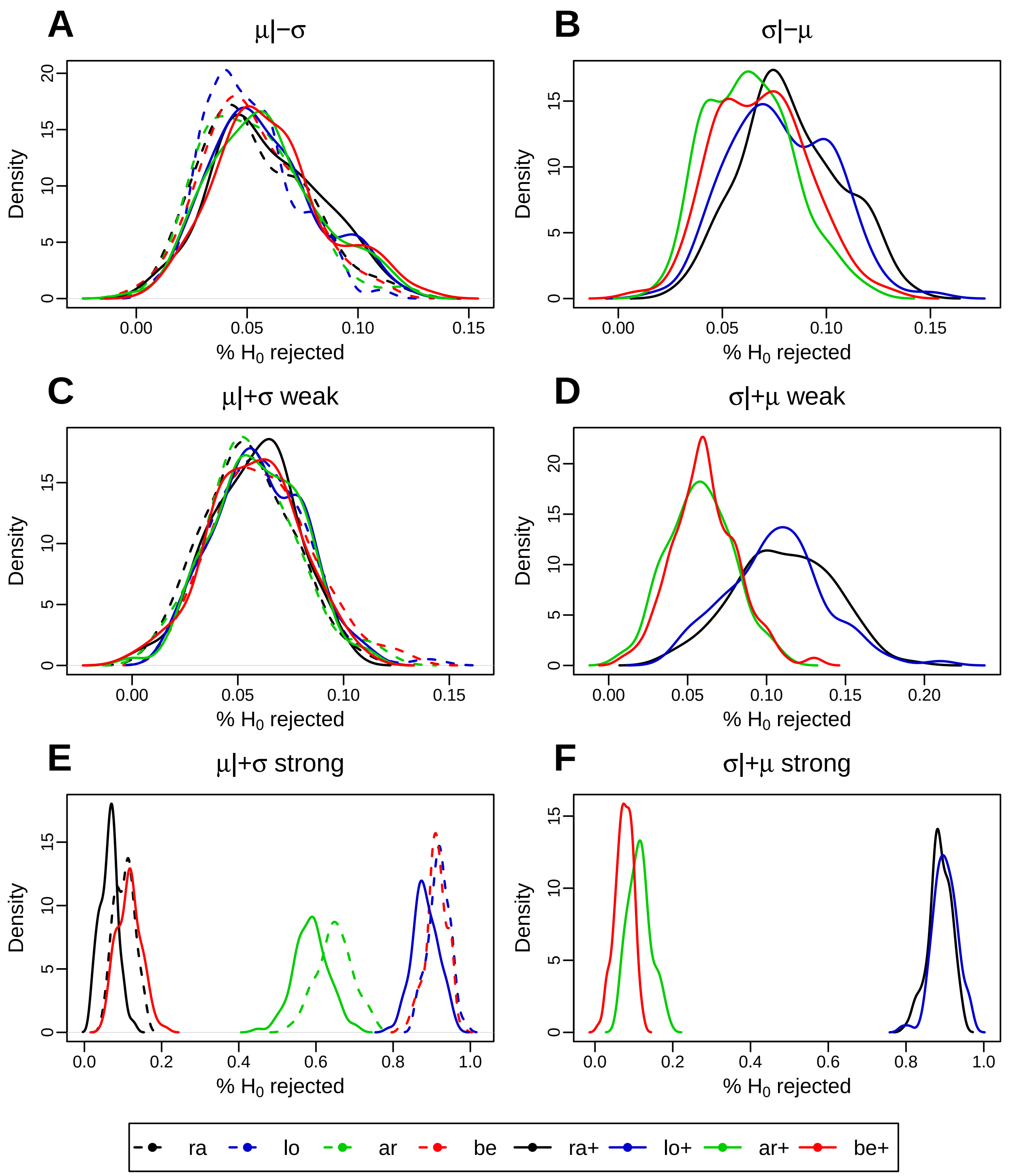

Figure S3: **Simulation study: Distribution of type I error rates of hypothesis tests for covariate effects on beta distributed methylation responses (n = 1763).** Kernel den sity estimates of estimated type I error rates are plotted across 100 sets of 100 CpG sites. The plots correspond to the average type I error rates shown in main text Figures 2 A and B in the main text. Settings are explained in Table 2 in the main text.

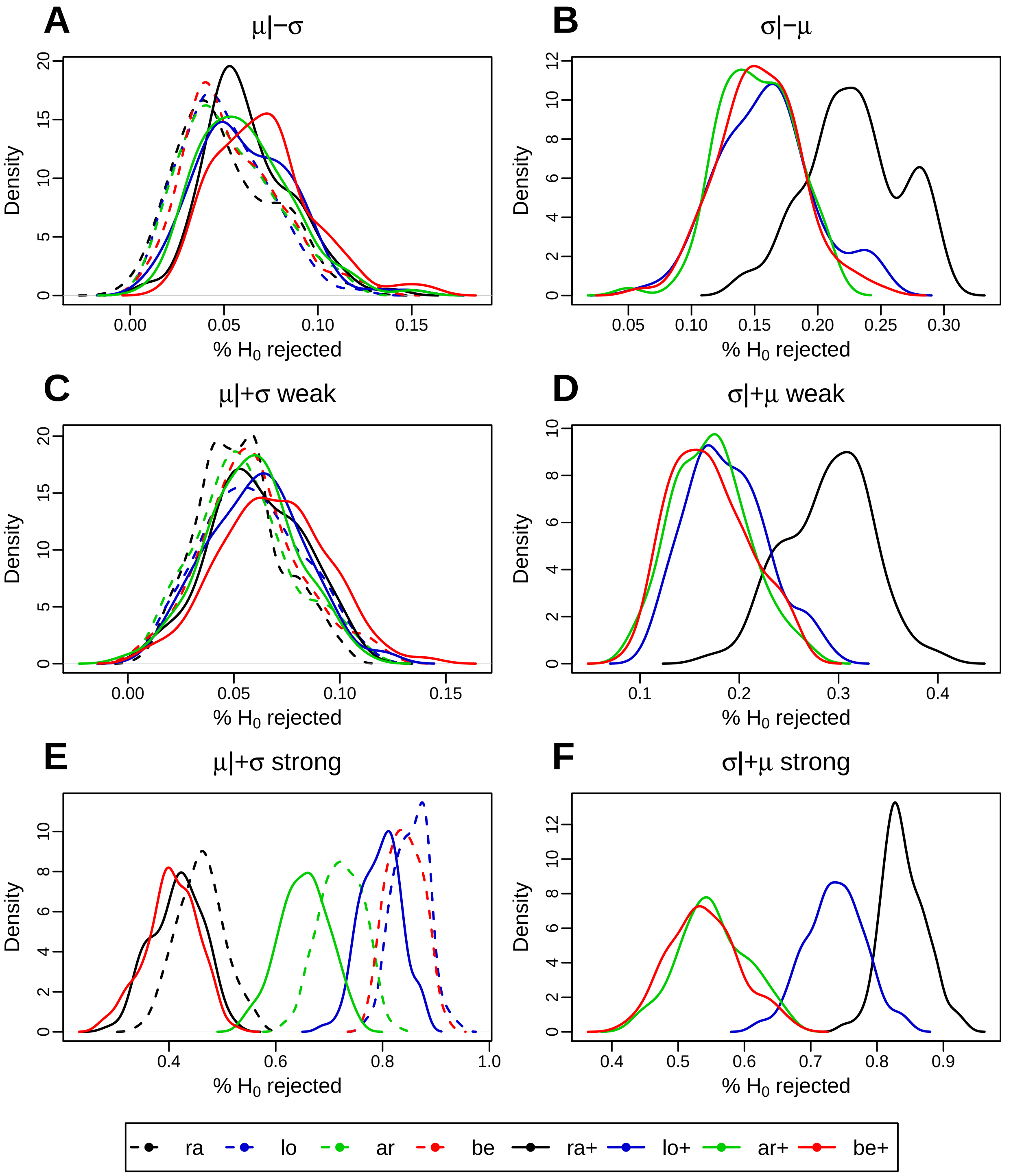

Figure S4: **Simulation study: Distribution of type I error rates of hypothesis tests for covariate effects on real-data distributed methylation responses (n = 1763).** Kernel density estimates of estimated type I error rates are plotted across 100 sets of 100 CpG sites. The plots correspond to the average type I error rates shown in Figures 2 C and D in the main text. Settings are explained in Table 2 in the main text.

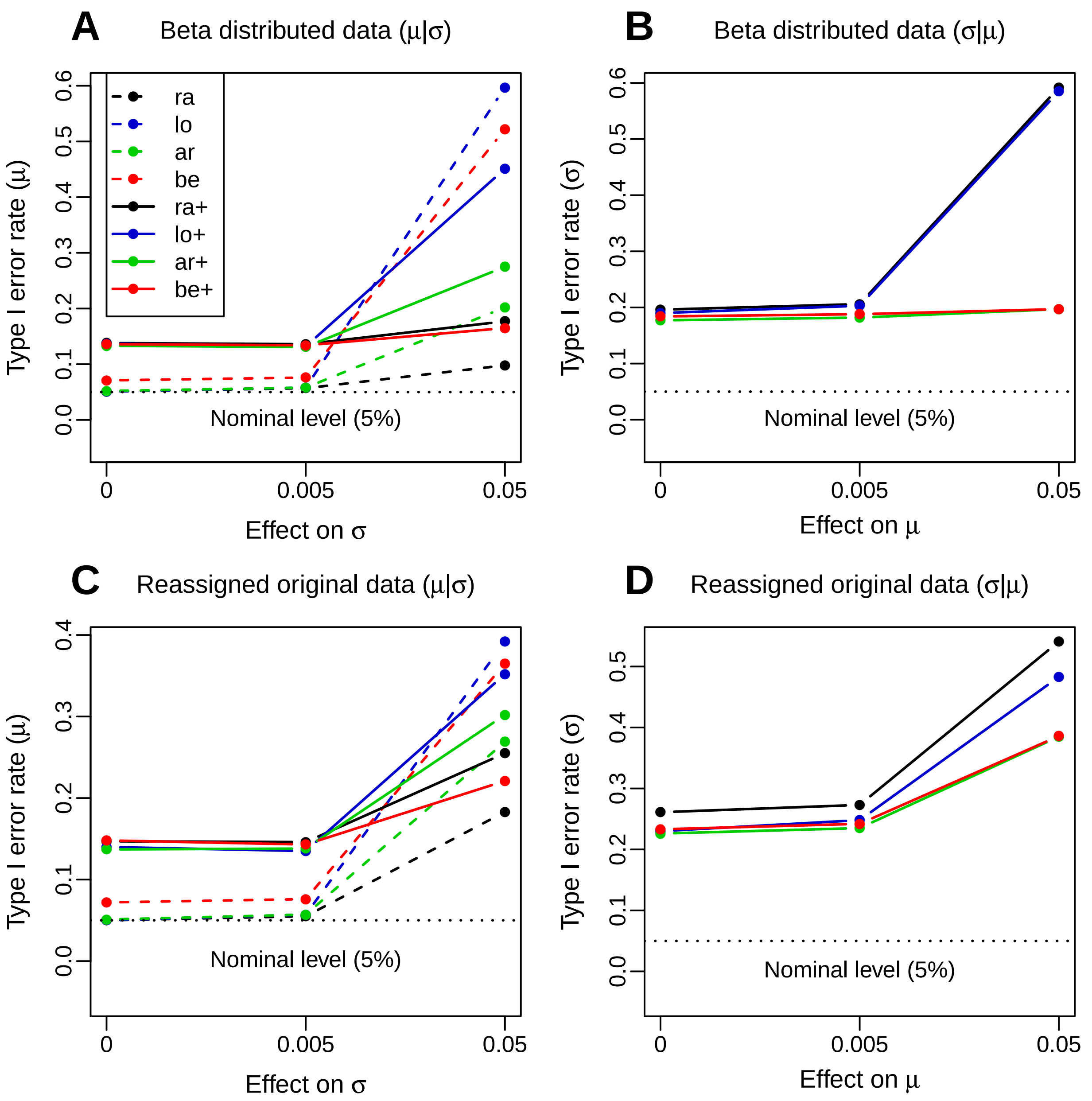

Figure S5: **Simulation study: Average estimated type I error rates of hypothesis tests for covariate effects (n = 250).** Average estimated type I error is plotted against effect size that the same covariate (BMI) had on the other distribution parameter. Simulation results are shown for beta distributed (**A, B**) and for real-data distributed methylation values (**C, D**). Model abbreviations are explained in Table 1 in the main text.

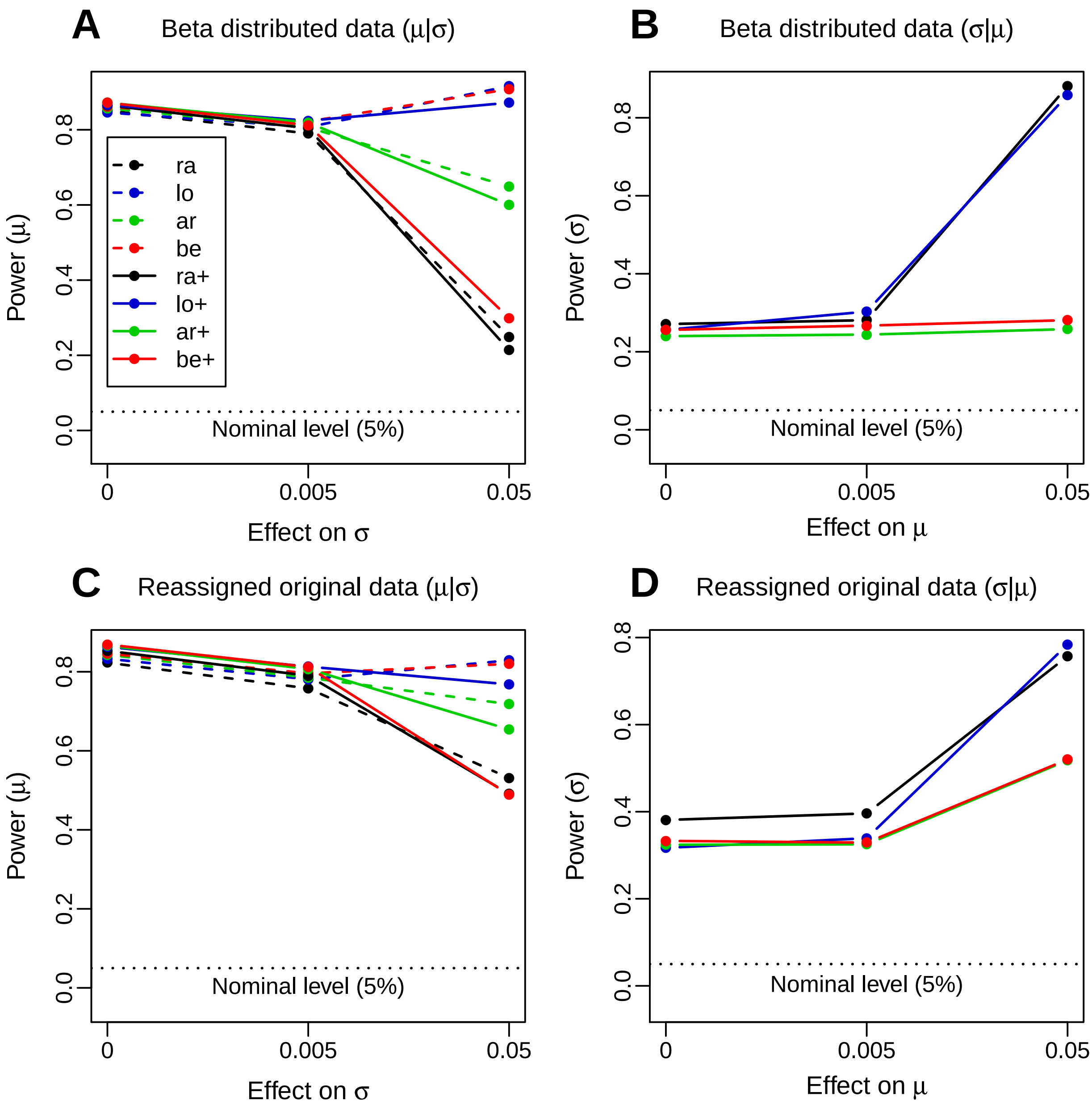

Figure S6: **Simulation study: Average estimated power of hypothesis tests for covariate effects ($\mathbf{n = 1763}$).** Average power is plotted against effect size that the same covariate (BMI) had on the other distribution parameter. Simulation results are shown for beta distributed (**A, B**) and for real-data distributed methylation values (**C, D**). Model abbreviations are explained in Table 1 in the main text.

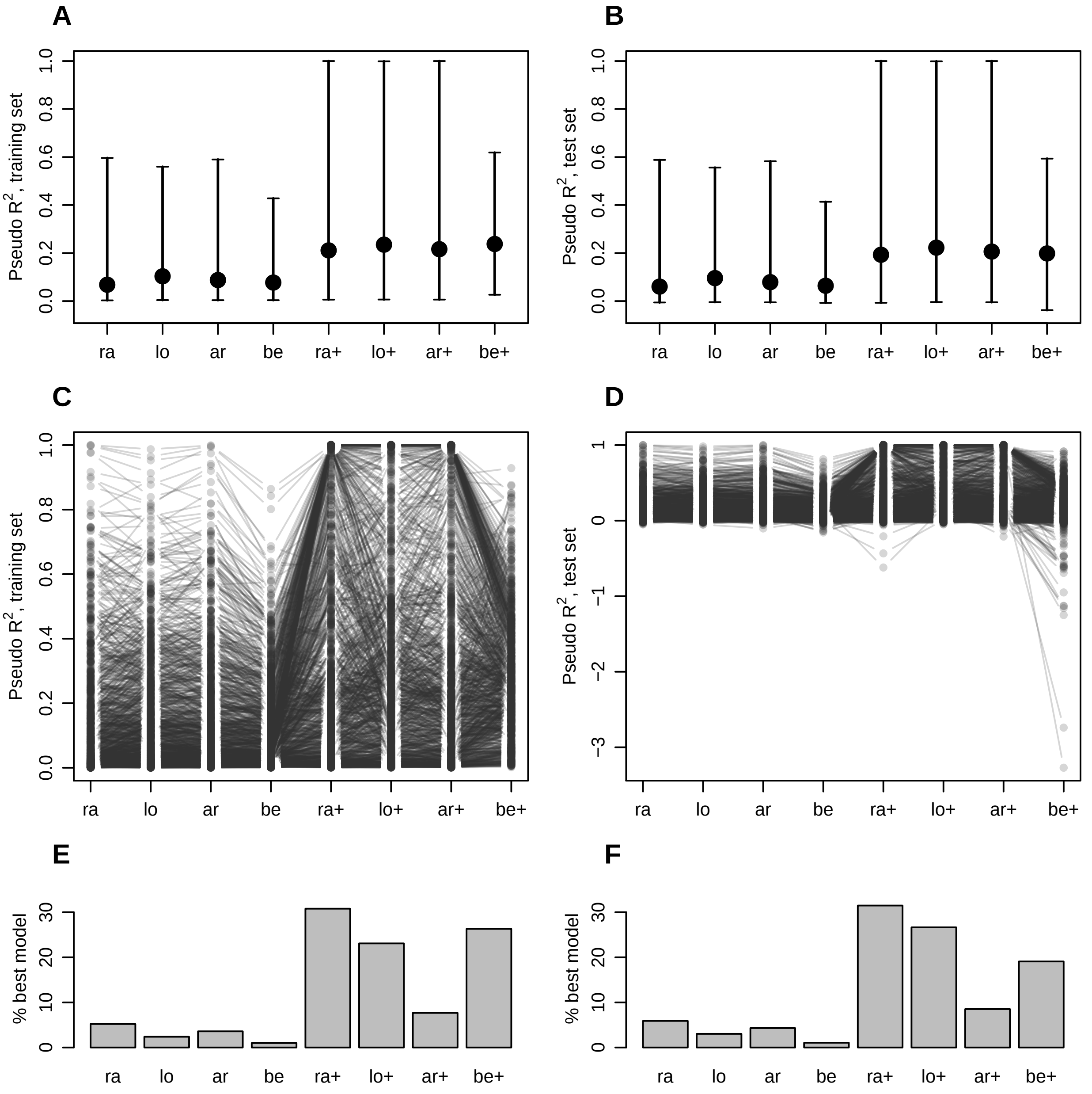

Figure S7: **Performance of competing models for DNA methylation data in a data set of acute lymphoblastic leukemia (ALL) patients and healthy controls (n = 695). A and B** Median, 5% and 95% quantile of pseudo R^2 in training and test data set, respectively, across the random set of the investigated CpG sites. **C and D** Pseudo R^2 values of individual CpG sites in training and test data set, respectively. 1000 CpG sites were randomly chosen for this plot. **D and E** Proportion of CpG sites for which the respective model had the largest pseudo R^2 measure as compared to the competing models, in training and test data set, respectively. Model abbreviations are explained in Table 1 in the main text.

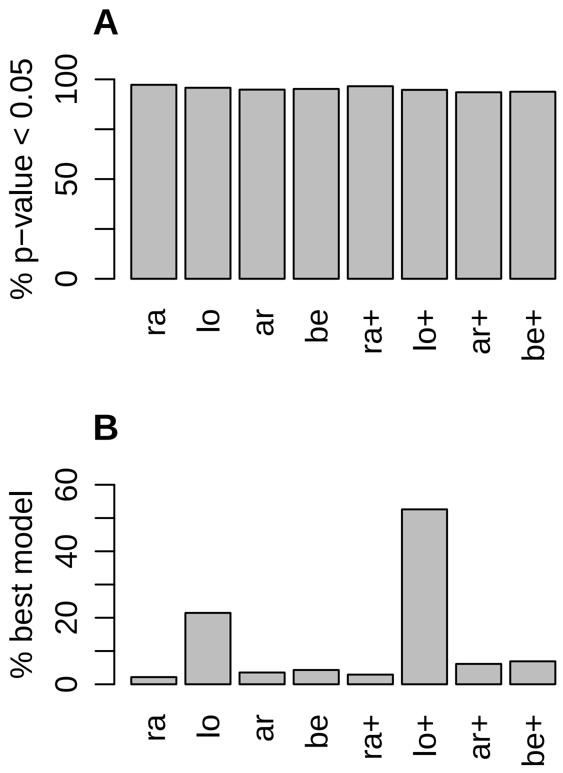

Figure S8: **Residual normal fit of competing models for DNA methylation data in a data set of acute lymphoblastic leukemia (ALL) patients and healthy controls (n = 695).** **A** Proportion of CpG sites for which significant deviation of residuals from normality was indicated by Shapiro-Wilk test p-value < 0.05. **B** Proportion of CpG sites for which the respective model had the best residual normal fit as compared to the competing models. Model abbreviations are explained in Table 1.

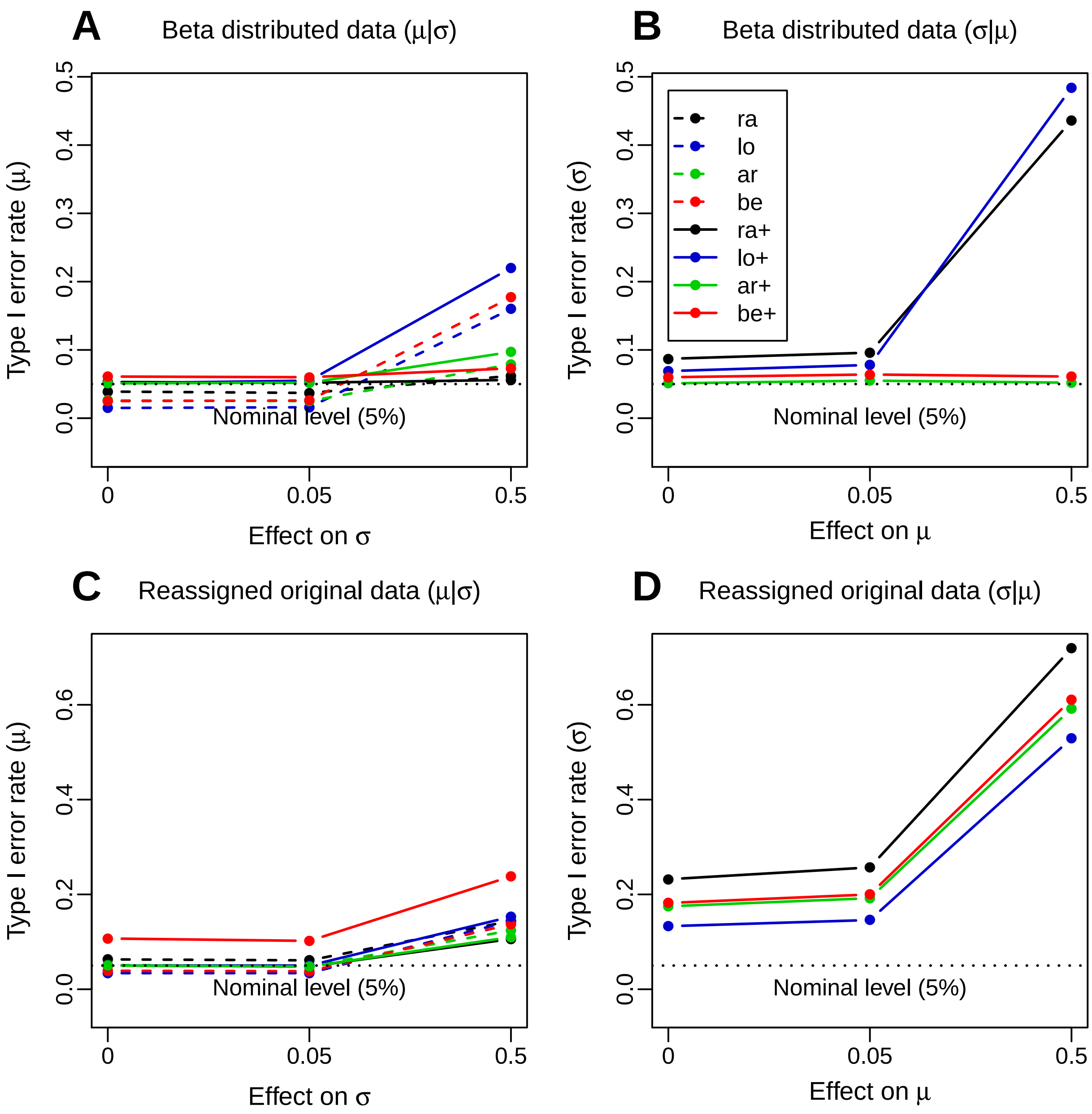

Figure S9: **Simulation study: Average estimated type I error rates of hypothesis tests for covariate effects (ALL data set; n = 695).** Average estimated type I error is plotted against effect size that the same covariate (T-ALL) had on the other distribution parameter. Simulation results are shown for beta distributed (**A, B**) and for real-data distributed methylation values (**C, D**). Model abbreviations are explained in Table 1 in the main text.

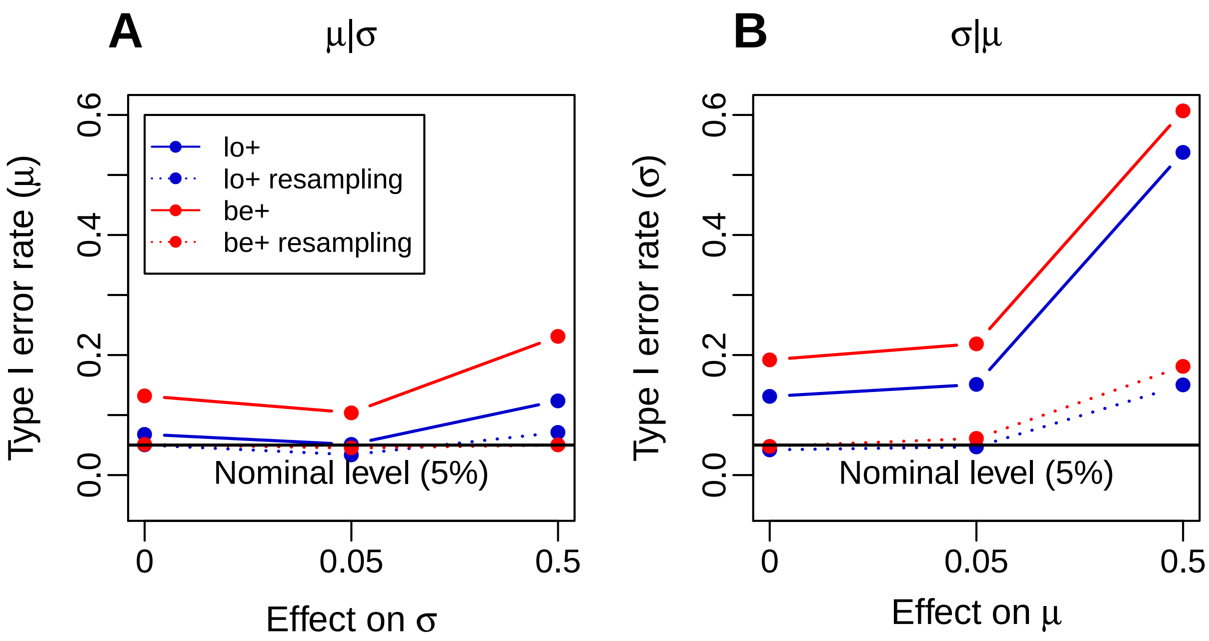

Figure S10: **Type I error control through the resampling procedure in a data set of acute lymphoblastic leukemia (ALL) patients and healthy controls (n = 695).** Observed type I error is plotted against effect size that the same covariate (T-ALL) had on the other distribution parameter. Simulation on real-data distributed methylation responses, before (solid lines) and after (dotdashed lines) application of the resampling procedure and inclusion of genetic variants as covariates. Model abbreviations are explained in Table 1.
